# Supplementary material for: Repetitive transcranial magnetic stimulation for depression after basal ganglia ischaemic stroke: protocol for a multicentre randomised double-blind placebo-controlled trial
Source: BMJ Open. 2018 Feb 3;8(2):e018011. doi: 10.1136/bmjopen-2017-018011 (PMC5829582; doi:10.1136/bmjopen-2017-018011)
Supplement: Author's manuscript [file bmjopen-2017-018011.draft_revisions.pdf]

BMJ Open is committed to open peer review. As part of this commitment we make the peer review history of every article we publish publicly available.

When an article is published we post the peer reviewers' comments and the authors' responses online. We also post the versions of the paper that were used during peer review. These are the versions that the peer review comments apply to.

The versions of the paper that follow are the versions that were submitted during the peer review process. They are not the versions of record or the final published versions. They should not be cited or distributed as the published version of this manuscript.

BMJ Open is an open access journal and the full, final, typeset and author-corrected version of record of the manuscript is available on our site with no access controls, subscription charges or pay-per-view fees (http://bmjopen.bmj.com).

If you have any questions on BMJ Open's open peer review process please email editorial.bmjopen@bmj.com

BMJ Open

Repetitive transcranial magnetic stimulation for depression after basal ganglia ischemic stroke: a multicenter, randomized, double-blind, placebo-controlled trial

Journal:	*BMJ Open*
Manuscript ID	bmjopen-2017-018011
Article Type:	Protocol
Date Submitted by the Author:	11-Jun-2017

Complete List of Authors:	Tang, Ying; Zhujiang Hospital of Southern Medical University,<br>Chen, Aimin; Zhujiang Hospital of Southern Medical University<br>Zhu, Shuzhen; Zhujiang Hospital of Southern Medical University<br>Yang, Li; Zhujiang Hospital of Southern Medical University<br>Zhou, Jiyuan; Southern Medical University, Department of Biostatistics, School of Public Health<br>Pan, Suyue; Southern Medical University Nanfang Hospital<br>Shao, Ming; Sichuan Bayi Rehabilitation Center<br>Zhao, Lianxu; Zhujiang Hospital of Southern Medical University

**Primary Subject Heading**:	Neurology
Secondary Subject Heading:	Mental health
Keywords:	repetitive transcranial magnetic stimulation, post-stroke depression, ischemic stroke

SCHOLARONE™ Manuscripts

For peer review only

Repetitive transcranial magnetic stimulation for depression after basal ganglia ischemic stroke: a multicenter, randomized, double-blind, placebo-controlled trial

Ying Tang[1], Aimin Chen[1], Shuzhen Zhu[1], Li Yang[1], Jiyuan Zhou[2], Suyue Pan[3], Min Shao[4], Lianxu Zhao[1]

For peer review only

ABSTRACT

Introduction: Studies suggest that repetitive transcranial magnetic stimulation (rTMS) is effective for treatment of depression and promotes the repair of white matter integrity. This study aims to assess the effectiveness of rTMS in treating depression after basal ganglia ischemic stroke and to examine whether such effects are related to restoration of white matter integrity.

Methods and analysis: Sixty-six participants will be recruited from Zhujiang Hospital, Nanfang Hospital and Sichuan Bayi Rehabilitation Hospital and randomized with a 1:1 ratio to receive active rTMS treatment or sham rTMS treatment in addition to routine supportive treatments. The data will be collected at 0, 2 and 4 weeks after the commencement of the treatment. The primary outcome is the measurement of 24-item Hamilton Depression Rating Scale (HAMD-24) scores, and the secondary outcomes include diffusion tensor imaging (DTI) results and the results of neuropsychological tests including National Institutes of Health Stroke Scale (NIHSS), Activities of Daily Living Scale (ADLs), Montreal Cognitive Assessment (MoCA), Aphsia Battery in Chinese (ABC), Social Support Revalued Scale (SSRS) and Medical Coping Questionnaire (MCMQ).

Ethics and dissemination: This study has been approved by the Ethics Committee of Zhujiang Hospital of Southern Medical University. The findings will be disseminated by publication in a peer-reviewed journal and by presentation in international conferences.

Trial registration number: ClinicalTrials.gov ID:NCT03159351.

Strengths and limitations of this study

1.Fill the research gaps of the effect of rTMS on PSD in China.

2.Explore the antidepressant effect of rTMS via a series of neuropsychological tests.

3.Put forward the hypothesis that the antidepressant effect of rTMS is related to its action on the LCSPT circuit and try to verify this hypothesis by Magnetic Resonance Imaging.

4.Incorporates mixed methods and double-blind.

5.Only performs a short-term treatment and follow-up(one month).

6.Only include two provinces' participants in China.

INTRODUCTION

Stroke is a global health problem and in 2015, the number of deaths related with stroke reached 6.326 million around the world, among which 45.89% were attributed to ischemic stroke[1]. Stroke survivors frequently suffered from depression[2] that severely affects the neurological function recovery of the patients[3]. The conventional treatments of post-stroke depression (PSD), including selective serotonin reuptake inhibitors (SSRIs)[4], tricyclic antidepressants (TCA)[5] and electroconvulsive therapy (ECT)[6], are not always effective. Recent evidence has shown that repetitive transcranial magnetic stimulation (rTMS) has antidepressant actions [7, 8] and produces comparable therapeutic effects to those of ECT[9]. According to Jorge et al, rTMS was effective and safe for treatment of PSD [10]. So far the exact mechanism underlying the antidepressant effect of rTMS remains unknown in spite of the various hypotheses that have been proposed[11-13].

The basal ganglia area is the predilection site of stroke. The basal ganglia area, which is not an exact anatomical concept but one originating from neuroimaging, consists of the basal ganglia, the capsula interna and the surrounding white matter [14]; in other words, the basal ganglia area covers most of the subcortical structures that constitute the limbic-cortical-striatal-pallidal-thalamic (LCSPT) circuit[15]. The limbic system is known to be closely related with emotional regulation[16], and as the LCSPT circuit is the most important part of the limbic system, LCSPT dysfunctions are thought to be associated with the occurrence of depression[17]. Investigators reported a high incidence of depression after cerebral infraction in the basal ganglia area[18]. Our group previously found that structural damage of the LCSPT circuit, as a result of white matter damage following cerebral infraction in the basal ganglia area, was associated with a high likeliness of PSD[19]. The nuclei in the LCSPT circuit are connected via nerve fibers, and damage of these nerve fibers disrupted the integrity of the white matter as shown by magnetic resonance imaging (MRI)[20]. As numerous studies have demonstrated the beneficial effect of rTMS treatment on white matter

For peer review only

integrity[21-23], we hypothesized that the antidepressant effect of rTMS is related to its action on the LCSPT circuit in patients with depression following cerebral infraction in the basal ganglia area.

To our knowledge, currently no studies have been reported to examine the effect of rTMS on PSD in China, and the mechanism underlying the effect of rTMS on PSD remains to be clarified. The aim of this multicenter randomized controlled trial is to assess the effectiveness of rTMS in treating PSD and investigate the role of the LCSPT circuit in the therapeutic effect of rTMS for PSD.

For peer review only

METHODS

Study design

This three-center, randomised, double blind, placebo controlled trial lasting for 36 months is designed to compare the therapeutic effects of active rTMS treatment and sham rTMS treatment in addition to routine supportive treatments in patients with PSD following cerebral infarction in the basal ganglia area. The routine supportive treatments include antiplatelet agents, blood pressure control, neurotrophic therapy, physical therapy, and cognitive rehabilitation prescribed based on the participants' condition. All the participants will receive psychiatric and neuropsychological tests and MRI examination at the time of enrollment and at the end of treatment. The study design is shown in the flow chart in **Figure 1**.

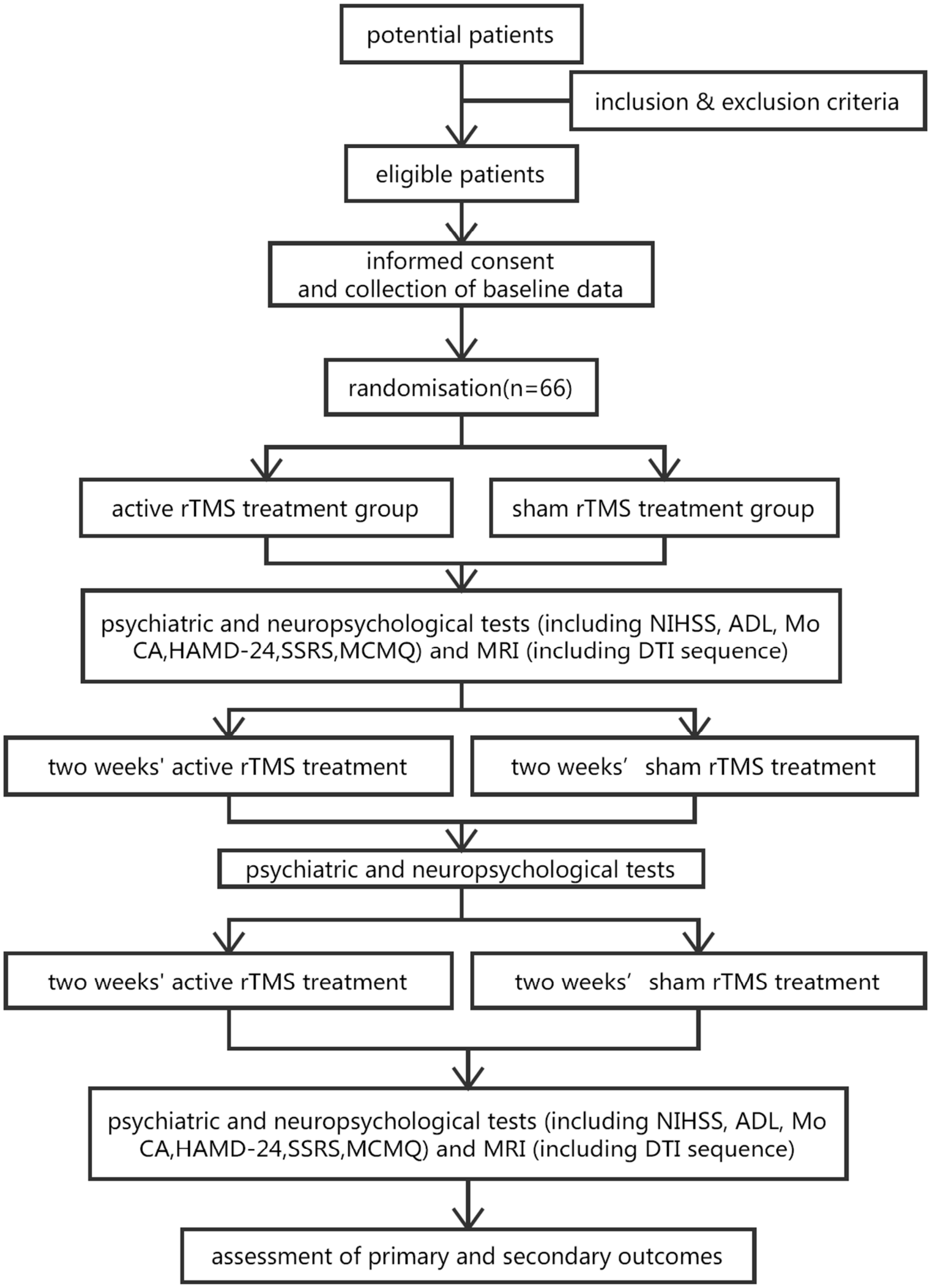

Figure 1 Flow chart of the study design.

Participant recruitment

The participants will be recruited by advertisement on Internet and using posters in

hospitals. The potential patients will be screened for enrollment according to the inclusion and exclusion criteria listed in Table 1.

Table 1 Inclusion and exclusion criteria

Criteria	Description
**Inclusion criteria**	1. First-time ischemic stroke with clinical and MRI or CT findings of basal ganglia stroke and a DSM-IV diagnosis of depression due to stroke (ICD-10-CM code 293.83[F06.32]);<br>2. Aged 25-75 years with a recent (from 3 weeks to 3 months) ischemic stroke;<br>3. Clear signs of neurological deficits in the acute phase;<br>4. Clear consciousness;<br>5. Right-handedness.
**Exclusion criteria**	1. Aphasia or severe cognitive impairment, severe hearing impairment, or severe language comprehension deficits due to other causes;<br>2. Other cerebral diseases such as Parkinson's disease, encephalitis, dementia, multiple sclerosis, head injury, ect.;<br>3. Severe systemic disease or ongoing neoplasia;<br>4. Ongoing post-operative recovery;<br>5. Prior history of depressive disorders or major trauma within 1 year, severe depression or any other severe mental disorders;<br>6. Addiction to drugs, alcohol or other substances;<br>7. Contraindications of MRI scan and rTMS treatment such as pacemaker implantation, a history of epilepsy, major head trauma, and seizures, ect;<br>8. Pregnant or breast-feeding women;

For peer review only

9. Participation in other clinical research projects;

10. Refusal to sign informed consent of this study.

Ethical considerations

This study has been approved by the Ethics Committee of Zhujiang Hospital of Southern Medical University. The methods, purpose and potential risks of this study will be fully explained to all the participants and their family members. Each patient will be asked to sign 3 informed consents before participating in this study.

Sample size calculation

The primary efficacy parameter is the change in HAMD-24 scores from the baseline to the end of the treatment. The sample size was calculated based on the data of Valliengo et al[24], who reported a response rate of 0.041 in patients receiving sham rTMS treatment and a rate of 0.375 in patients having active rTMS treatment. Considering the low efficiency of sham rTMS treatment, we calculated the sample size using the following formula:

$$n_1 = n_2 = \frac{\left(Z_{\alpha/2} + Z_{\beta}\right)^2}{2\left(\sin^{-1}\sqrt{p_2} - \sin^{-1}\sqrt{p_1}\right)^2}。$$

The result showed that a sample size of 26 in each group was sufficient by assuming a two-tailed significance level of 5% and a statistical power of 90%. We estimate that the dropout rate will be less than 25%, and considering the need of randomisation, we assume that at least 33 participants are required in each group.

Randomisation and blinding

For randomisation of the patients, random numbers will be generated using SAS9.4 software by statisticians based on a randomized block design with a block length of 6. The magnetic stimulation coils for active and sham treatments have identical appearances and will be numbered by a statistician. The random numbers will be printed and sent to each trial center in sealed envelopes. Upon enrollment of an eligible patient, the investigator opens the envelope to receive the number of the magnetic stimulation coil to be used, without knowing whether the patient receives active or sham treatment. In this double-blind trial, both the participants and investigator are blinded to the nature of treatment (active or sham).

Intervention and comparison

For peer review only

All the participants will receive active or sham rTMS treatment in addition to routine supportive treatments including antiplatelet agents, blood pressure control, neurotrophic therapy, physical therapy, and cognitive rehabilitation prescribed based on the condition of the individual participant.

rTMS treatment

The protocols of active rTMS treatment have been established by experts after thorough discussions and the detailed parameters are listed in **Table 2.**

Table 2 Active rTMS treatment protocols

Localization	Frequency	Intensity	Times/ train	trains	Duration	Total times
Left DLPFC	10 Hz	110%MT	200	10	40 seconds	20
Right DLPFC	1 Hz	100%MT	30	10	10 seconds	20

DLPFC: Dorsolateral prefrontal cortex; MT: Motor threshold.

Magnetic resonance imaging

Upon enrollment and at the end of this trial, the patient will receive MRI examination using a 3.0T PHILIPS Magnetic Trio Tim scanner. For the standard workup, MRI will be performed using the following scan parameters for different sequences: for T1-weighted sequence, time of echo (TE)=20 ms, time of repetition (TR)=2000 ms, number of signal averaged (NSA)=1, slice=32, slice thickness=3 mm; for T2-weighted sequence, TE=80 ms, TR=3000 ms, NSA=2, slice=32, slice thickness=3 mm; for FLAIR sequence, TE=80 ms, TR=3000 ms, NSA=2, slice=32, slice thickness=3 mm; for diffusion tensor imaging (DTI) sequence: TE=68 ms, TR=2300 ms, NSA=1, slice=32, slice thickness=3 mm, b_0=0, b_1=1000, matrix=512*512. The nerve fiber tracks in the basal ganglia area are revealed by three-dimensional imaging derived from the 4 image sets.

Outcome assessment

The assessment data will be collected at baseline, 2 and 4 weeks after commencement

For peer review only

of the treatment, and 4 weeks after completion of the treatment.

Baseline assessment

The demographic data will be collected from the patients, including age, gender, height, body weight, education level, occupation, and marital status. The vital signs will be measured including body temperature, pulse, respiration, and blood pressure. The data including the past history and treatment prescription, levels of blood lipids and blood glucose, liver function index and renal function index will be collected before randomisation by reviewing the medical records of the patients.

Primary efficacy parameters

We plan to measure the response rate and remission rate as primary efficacy parameters, namely the changes in HAMD-24 scores from baseline to the end of the treatment. The response to treatment is defined as at least a 50% reduction of the HAMD-24 total score with a final HAMD-24 score below 9. Remission is defined as a reduction of the HAMD-24 total score by at least 50% from the baseline. As a valid and reliable scale for assessing the severity of depression, HAMD scale has been widely used in studies of PSD and shows a good sensitivity in assessing treatment responses. HAMD-24 scale consists of subscales that examine interest, anxiety, hopelessness, sleep status, and self-abasement, etc., and a higher score suggests a poorer condition of mental disorder[25]. HAMD-24 scale will be scored for 3 times at 0, 2, and 4 weeks after the commencement of treatment.

Secondary outcome measures

DTI results

Nerve fibers are important components of the LCSPT circuit apart from the cerebral cortex, corpus striatum and thalami. The nuclei are connected by abundant nerve fibers. DTI is a special sequence of MRI, which is currently the best non-invasive method to observe and track the nerve tracts. We will assess the changes of the nerve fibers based on the findings of the DTI parameters including the fractional anisotropy (FA), apparent diffusion coefficient (ADC), neural fiber number (NFN).

Neuropsychological tests

The neuropsychological status of the patients will be examined using the following scales or questionnaires: National Institutes of Health Stroke Scale (NIHSS), Activities of Daily Living Scale (ADLs), Montreal Cognitive Assessment (MoCA), which are widely used to evaluate neurological functions; Aphsia Battery in Chinese (ABC), which is used to estimate the linguistic functions of Chinese patients; and

For peer review only

Social Support Revalued Scale (SSRS) and Medical Coping Questionnaire score (MCMQ), which evaluate how social support influences emotions of the patient. All the tests will be implemented at 0, 2 and 4 weeks after the commencement of the treatment.

Assessment of adverse events

All the adverse events (AEs) will be reported and recorded in the case report form (CRF) by investigators. The investigators will record the date of the occurrence, duration, and severity of the AEs, along with the treatments and consequences. Serious AEs will be reported to the project leader and the ethics committee immediately, and the latter will evaluate whether the study should proceed. Those participants who suffer harm from this trial will be provided with compensation.

Quality control and data management

The trial protocol has been revised for several times by experts in neurology, rehabilitation, medical imaging, statistics and psychology. All the investigators have participated in the training sessions to ensure that they fully understand the trial protocols and the standard procedures before the study. Inspectors will be nominated to regularly visit each research center to monitor the implementation of the protocols and assess the compliance of the participants and investigators. All the data will be recorded on CRF by the investigators. A staff member will be appointed to manage all the CRFs and ensure the completeness of the data. The data will be double-entered by statisticians to ensure the accuracy of the data. Statisticians in our team will be responsible for data management and analysis.

Statistical analysis

The quantitative data, including the demographic indicators, laboratory test result, MRI results and neuropsychological test results, will be expressed as numbers, means with standard deviation or median with range or quartile range as appropriate. These data will be compared using independent-sample *t* test or Rank sum test depending on whether the data are normally distributed. The qualitative data, including the response and emission rates and incidences of the AEs, will be expressed as frequency or frequency rate and compared using χ^2 test between the two groups. All the data will be double-entered into Epidata software and analysed using SAS software by statisticians blinded to the participants' allocation. A *P* value less than 0.05 will be considered to indicate a statistically significant difference.

DISCUSSION

For peer review only

Studies indicate that rTMS is effective in the treatment of depression[10] and promotes the repair of white matter integrity[21–23]. This trial is expected to produce additional evidence to verify the effects of rTMS treatment on depression after ischemic stroke in the basal ganglia area and to explore the role of the LCSPT circuit in PSD. Findings from this study will fill the gaps in randomized controlled trials of rTMS for PSD in China, and contribute to the clinical evaluation of rTMS for PSD treatment and the improvement of the neural loop theory.

Author affiliations

[1]Zhujiang Hospital of Southern Medical University, Guangzhou, Guangdong, China

[2]School of Public Health, Southern Medical University, Guangzhou, Guangdong, China

[3]Nanfang Hospital of Southern Medical University, Guangzhou, Guangdong, China

[4]Sichuan Bayi Rehabilitation Center, Chengdu, Sichuan, China

Acknowledgements Authors appreciate the effort of all people participating in this trail.

Contributors Shuzhen Z, Suyue P, Ming S and Lianxu Z participated in the conception and design of the trial. Aimin C, Jiyuan Z ,and Lianxu Z participated in planning the annalysis of the data. Ying T, Aimin C, Li Y ,paticipated in data collection, particitants recruitment. Aimin C, Li Y, Suyue P, Ming S and Lianxu Z participated in the treatment of particitants. Ying T and Lianxu Z participated in drafting the manuscript. All the authors discussed,revised and approved the final manuscript.

Correspondence to Lianxu Zhao; E-mail: zhaolianxu@smu.edu.cn

Funding This study was supported by grants of the Training Program of the Clinical Research Plan of Southern Medical University (LC2016PY031).

Competing interests None declared.

Patient consent Obtained Obtained.

Ethics approval The ethics committees of Zhujiang Hospital of Southern Medical University approved the study.

Provenance and peer review Not commissioned; peer reviewed for ethical and

For peer review only

funding approval prior to submission.

Data sharing statement The results of this original research will be disseminated by publication in a peer-reviewed journal and presentation at international conferences.

Trial status The trial is currently ongoing in the stage of participant enrollment; the recruitment of patients started on 1 June, 2017 and will be completed in 31 May, 2020.

Open Access This is an Open Access article distributed in accordance with the terms of the Creative Commons Attribution (CC BY 4.0) license, which permits others to distribute, remix, adapt and build upon this work, for commercial use, provided the original works is properly cited. See: http://creativecommons.org/licenses/by/4.0/

REFERENCES

References:

1 Global, regional, and national life expectancy, all-cause mortality, and cause-specific mortality for 249 causes of death, 1980-2015: a systematic analysis for the Global Burden of Disease Study 2015. *LANCET*. 2016; **388**: 1459-544.

2 Chatterjee K, Fall S, Barer D. Mood after stroke: a case control study of biochemical, neuro-imaging and socio-economic risk factors for major depression in stroke survivors. *BMC NEUROL*. 2010; **10**: 125.

3 Matsuzaki S, Hashimoto M, Yuki S, Koyama A, Hirata Y, Ikeda M. The relationship between post-stroke depression and physical recovery. *J Affect Disord*. 2015; **176**: 56-60.

4 Mead GE, Hsieh CF, Hackett M. Selective serotonin reuptake inhibitors for stroke recovery. *JAMA*. 2013; **310**: 1066-67.

5 Capaldi VN, Wynn GH. Post stroke depression: treatments and complications in a young adult. *Psychiatr Q*. 2010; **81**: 73-79.

6 Berggren A, Gustafson L, Hoglund P, Johanson A. A long-term follow-up of clinical response and regional cerebral blood flow changes in depressed patients treated with ECT. *J Affect Disord*. 2014; **167**:

For peer review only

235-43.

7 Kim BR, Kim DY, Chun MH, Yi JH, Kwon JS. Effect of repetitive transcranial magnetic stimulation on cognition and mood in stroke patients: a double-blind, sham-controlled trial. *Am J Phys Med Rehabil*. 2010; **89**: 362-68.

8 Levkovitz Y, Isserles M, Padberg F, Lisanby SH, Bystritsky A, Xia G, *et al.*. Efficacy and safety of deep transcranial magnetic stimulation for major depression: a prospective multicenter randomized controlled trial. *WORLD PSYCHIATRY*. 2015; **14**: 64-73.

9 Ren J, Li H, Palaniyappan L, Liu H, Wang J, Li C, *et al.*. Repetitive transcranial magnetic stimulation versus electroconvulsive therapy for major depression: a systematic review and meta-analysis. *Prog Neuropsychopharmacol Biol Psychiatry*. 2014; **51**: 181-89.

10 Jorge RE, Robinson RG, Tateno A, Narushima K, Acion L, Moser D, *et al.*. Repetitive transcranial magnetic stimulation as treatment of poststroke depression: a preliminary study. *Biol Psychiatry*. 2004; **55**: 398-405.

11 Yulug B, Hanoglu L, Tavli AM, Yilmaz NH, Kilic E. The Brain Protective Effect of rTMS (Repetitive Transcranial Magnetic Stimulation) in Depression: A Mini-Review in Animal Studies. *MED CHEM*. 2016; **12**: 500-05.

12 Wang HN, Wang L, Zhang RG, Chen YC, Liu L, Gao F, *et al.*. Anti-depressive mechanism of repetitive transcranial magnetic stimulation in rat: the role of the endocannabinoid system. *J PSYCHIATR RES*. 2014; **51**: 79-87.

13 Peng H, Zheng H, Li L, Liu J, Zhang Y, Shan B, *et al.*. High-frequency rTMS treatment increases white matter FA in the left middle frontal gyrus in young patients with treatment-resistant depression. *J Affect Disord*. 2012; **136**: 249-57.

14 Naritomi H, Sawada T, Kuriyama Y, Kinugawa H, Kaneko T, Takamiya M. Effect of chronic middle cerebral artery stenosis on the local cerebral hemodynamics. *STROKE*. 1985; **16**: 214-19.

15 Haber SN. The place of dopamine in the cortico-basal ganglia circuit. *NEUROSCIENCE*. 2014; **282**: 248-57.

16 Joyce JN. The dopamine hypothesis of schizophrenia: limbic interactions with serotonin and norepinephrine. *Psychopharmacology (Berl)*. 1993; **112**: S16-34.

17 Berton O, Nestler EJ. New approaches to antidepressant drug discovery: beyond monoamines. *NAT REV NEUROSCI*. 2006; **7**: 137-51.

18 Wu RH, Li Q, Tan Y, Liu XY, Huang J. Depression in silent lacunar infarction: a cross-sectional study of its association with location of silent lacunar infarction and vascular risk factors. *NEUROL SCI*.

For peer review only

2014; **35**: 1553-59.

19 涂加善, 刘振华, 黄凡衡, 陈爱敏, 蔡卫卫, 祝淑贞, *et al.*. 磁共振弥散张量成像对基底节区梗死后抑郁状况的评估. *中华神经科杂志*. 2012; **45**: 590-94.

20 Nugent AC, Davis RM, Zarate CJ, Drevets WC. Reduced thalamic volumes in major depressive disorder. *Psychiatry Res*. 2013; **213**: 179-85.

21 Kozel FA, Johnson KA, Nahas Z, Nakonezny PA, Morgan PS, Anderson BS, *et al.*. Fractional anisotropy changes after several weeks of daily left high-frequency repetitive transcranial magnetic stimulation of the prefrontal cortex to treat major depression. *J ECT*. 2011; **27**: 5-10.

22 Yang W, Liu TT, Song XB, Zhang Y, Li ZH, Cui ZH, *et al.*. Comparison of different stimulation parameters of repetitive transcranial magnetic stimulation for unilateral spatial neglect in stroke patients. *J NEUROL SCI*. 2015; **359**: 219-25.

23 Allendorfer JB, Storrs JM, Szaflarski JP. Changes in white matter integrity follow excitatory rTMS treatment of post-stroke aphasia. *Restor Neurol Neurosci*. 2012; **30**: 103-13.

24 Valiengo LC, Goulart AC, de Oliveira JF, Bensenor IM, Lotufo PA, Brunoni AR. Transcranial direct current stimulation for the treatment of post-stroke depression: results from a randomised, sham-controlled, double-blinded trial. *J Neurol Neurosurg Psychiatry*. 2016.

25 Williams JB, Kobak KA, Bech P, Engelhardt N, Evans K, Lipsitz J, *et al.*. The GRID-HAMD: standardization of the Hamilton Depression Rating Scale. *Int Clin Psychopharmacol*. 2008; **23**: 120-29.

For peer review only

CONSORT 2010 checklist of information to include when reporting a randomised trial*

Section/Topic	Item No	Checklist item	Reported on page No
**Title and abstract**			
	1a	Identification as a randomised trial in the title	1
	1b	Structured summary of trial design, methods, results, and conclusions (for specific guidance see CONSORT for abstracts)	1
**Introduction**			
Background and objectives	2a	Scientific background and explanation of rationale	1,2
	2b	Specific objectives or hypotheses	3
**Methods**			
Trial design	3a	Description of trial design (such as parallel, factorial) including allocation ratio	2
	3b	Important changes to methods after trial commencement (such as eligibility criteria), with reasons	
Participants	4a	Eligibility criteria for participants	4
	4b	Settings and locations where the data were collected	1
Interventions	5	The interventions for each group with sufficient details to allow replication, including how and when they were actually administered	5
Outcomes	6a	Completely defined pre-specified primary and secondary outcome measures, including how and when they were assessed	6
	6b	Any changes to trial outcomes after the trial commenced, with reasons	
Sample size	7a	How sample size was determined	4
	7b	When applicable, explanation of any interim analyses and stopping guidelines	
Randomisation:			
Sequence generation	8a	Method used to generate the random allocation sequence	
	8b	Type of randomisation; details of any restriction (such as blocking and block size)	5
Allocation concealment mechanism	9	Mechanism used to implement the random allocation sequence (such as sequentially numbered containers), describing any steps taken to conceal the sequence until interventions were assigned	5
Implementation	10	Who generated the random allocation sequence, who enrolled participants, and who assigned participants to interventions	5
Blinding	11a	If done, who was blinded after assignment to interventions (for example, participants, care providers, those	5

For peer review only - http://bmjopen.bmj.com/site/about/guidelines.xhtml

		assessing outcomes) and how	
	11b	If relevant, description of the similarity of interventions	
Statistical methods	12a	Statistical methods used to compare groups for primary and secondary outcomes	7
	12b	Methods for additional analyses, such as subgroup analyses and adjusted analyses	
**Results**			
Participant flow (a diagram is strongly recommended)	13a	For each group, the numbers of participants who were randomly assigned, received intended treatment, and were analysed for the primary outcome	
	13b	For each group, losses and exclusions after randomisation, together with reasons	
Recruitment	14a	Dates defining the periods of recruitment and follow-up	
	14b	Why the trial ended or was stopped	
Baseline data	15	A table showing baseline demographic and clinical characteristics for each group	
Numbers analysed	16	For each group, number of participants (denominator) included in each analysis and whether the analysis was by original assigned groups	
Outcomes and estimation	17a	For each primary and secondary outcome, results for each group, and the estimated effect size and its precision (such as 95% confidence interval)	
	17b	For binary outcomes, presentation of both absolute and relative effect sizes is recommended	
Ancillary analyses	18	Results of any other analyses performed, including subgroup analyses and adjusted analyses, distinguishing pre-specified from exploratory	
Harms	19	All important harms or unintended effects in each group (for specific guidance see CONSORT for harms)	
**Discussion**			
Limitations	20	Trial limitations, addressing sources of potential bias, imprecision, and, if relevant, multiplicity of analyses	
Generalisability	21	Generalisability (external validity, applicability) of the trial findings	7
Interpretation	22	Interpretation consistent with results, balancing benefits and harms, and considering other relevant evidence	7
**Other information**			
Registration	23	Registration number and name of trial registry	1
Protocol	24	Where the full trial protocol can be accessed, if available	8
Funding	25	Sources of funding and other support (such as supply of drugs), role of funders	8

*We strongly recommend reading this statement in conjunction with the CONSORT 2010 Explanation and Elaboration for important clarifications on all the items. If relevant, we also recommend reading CONSORT extensions for cluster randomised trials, non-inferiority and equivalence trials, non-pharmacological treatments, herbal interventions, and pragmatic trials. Additional extensions are forthcoming: for those and for up to date references relevant to this checklist, see www.consort-statement.org.

For peer review only - http://bmjopen.bmj.com/site/about/guidelines.xhtml

For peer review only

BMJ Open

Repetitive transcranial magnetic stimulation for depression after basal ganglia ischemic stroke: protocol for a multicenter, randomized, double-blind, placebo-controlled trial

Journal:	*BMJ Open*
Manuscript ID	bmjopen-2017-018011.R1
Article Type:	Protocol
Date Submitted by the Author:	13-Sep-2017

Complete List of Authors:	Tang, Ying; Zhujiang Hospital of Southern Medical University,<br>Chen, Aimin; Zhujiang Hospital of Southern Medical University<br>Zhu, Shuzhen; Zhujiang Hospital of Southern Medical University<br>Yang, Li; Zhujiang Hospital of Southern Medical University<br>Zhou, Jiyuan; Southern Medical University, Department of Biostatistics, School of Public Health<br>Pan, Suyue; Southern Medical University Nanfang Hospital<br>Shao, Ming; Sichuan Bayi Rehabilitation Center<br>Zhao, Lianxu; Zhujiang Hospital of Southern Medical University

**Primary Subject Heading**:	Neurology
Secondary Subject Heading:	Mental health
Keywords:	repetitive transcranial magnetic stimulation, post-stroke depression, ischemic stroke

SCHOLARONE™ Manuscripts

For peer review only

Repetitive transcranial magnetic stimulation for depression after basal ganglia ischemic stroke: protocol for a multicenter, randomized, double-blind, placebo-controlled trial

Ying Tang[1], Aimin Chen[2], Shuzhen Zhu[3], Li Yang[4], Jiyuan Zhou[5], Suyue Pan[6], Min Shao[7], Lianxu Zhao[8*]

Author information:

1 Zhujiang Hospital of Southern Medical University, Guangzhou, Guangdong, China. E-mail: tangying05@qq.com.

2 Zhujiang Hospital of Southern Medical University, Guangzhou, Guangdong, China. E-mail: doctoramchen@163.com.

3 Zhujiang Hospital of Southern Medical University, Guangzhou, Guangdong, China. E-mail: miraclezsz@qq.com.

4 Zhujiang Hospital of Southern Medical University, Guangzhou, Guangdong, China. E-mail: ylsnn09@163.com.

5 School of Public Health, Southern Medical University, Guangzhou, Guangdong, China. E-mail: zhoujiyuan5460@hotmail.com.

6 Nanfang Hospital of Southern Medical University, Guangzhou, Guangdong, China. E-mail: pansuyue82@qq.com.

7 Sichuan Bayi Rehabilitation Center, Chengdu, Sichuan, China. E-mail: 1961523453@qq.com.

8 Zhujiang Hospital of Southern Medical University, Guangzhou, Guangdong, China.

*Corresponding author, postal address: No.253, Industrial Avenue, Zhujiang Hospital of Southern Medical University, Guangzhou, Guangdong, China, 510280, E-mail: zhaolianxu@smu.edu.cn, phone: +86 2062783082.

Word count: 3311 words.

ABSTRACT

Introduction: Studies suggest that repetitive transcranial magnetic stimulation (rTMS) is effective for treatment of depression and promotes the repair of white matter integrity. This study aims to assess the effectiveness of rTMS in treating depression after basal ganglia ischemic stroke and to examine whether such effects are related to restoration of white matter integrity.

Methods and analysis: Sixty-six participants will be recruited from Zhujiang Hospital, Nanfang Hospital and Sichuan Bayi Rehabilitation Hospital and randomized with a 1:1 ratio to receive active rTMS treatment or sham rTMS treatment in addition to routine supportive treatments. The data will be collected at 0, 2 and 4 weeks after the commencement of the treatment. The primary outcome is the measurement of 24-item Hamilton Depression Rating Scale (HAMD-24) scores, and the secondary outcomes include diffusion tensor imaging (DTI) results and the results of neuropsychological tests including National Institutes of Health Stroke Scale (NIHSS), Activities of Daily Living Scale (ADLs), Montreal Cognitive Assessment (MoCA),

For peer review only

Clinical Global Impressions scales(CGI), Aphsia Battery in Chinese (ABC), Social Support Revalued Scale (SSRS) and Medical Coping Questionnaire (MCMQ).

Ethics and dissemination: This study has been approved by the Ethics Committee of Zhujiang Hospital of Southern Medical University. The findings will be disseminated by publication in a peer-reviewed journal and by presentation in international conferences.

Trial registration: ClinicalTrials.gov, Identifier: NCT03159351.

Keywords: repetitive transcranial magnetic stimulation (rTMS), post-stroke depression (PSD), ischemic stroke.

Strengths and limitations of this study

- The findings of this study will fill the research gaps of the effect of rTMS on PSD in China.
- This study will explore the antidepressant effect of rTMS via a series of neuropsychological tests.
- This study will test the hypothesis that the antidepressant effect of rTMS is related to its action on the LCSPT circuit based on evidence from magnetic resonance imaging findings.
- The patients will receive only short-term treatment and will be followed up for 1 month.
- Participants from only two provinces in China will be included.

INTRODUCTION

Background and rationale

Stroke is a global health problem and in 2015, the number of deaths related with stroke reached 6.326 million around the world, among which 45.89% were attributed to ischemic stroke[1]. Depression occurs frequently in the stroke survivors and has a significant impact on post-stroke rehabilitation[2]. The conventional treatment of post-stroke depression (PSD) relies on supportive therapy and medications, but a proportion of patients can remain treatment-refractory[3]. Studies has shown that repetitive transcranial magnetic stimulation (rTMS) has a definite antidepressant effect on major depression[4], and a recent meta-analysis also suggests the beneficial effects of rTMS on PSD, but such studies should be carefully examined for their heterogeneity and potential biases[5]. To our knowledge, currently no studies have been reported to examine the effect of rTMS on PSD in China, and the mechanism by which rTMS improves PSD remains to be clarified. In addition, the stimulation parameter settings in rTMS treatment still awaits standardization, and it is difficult to determine the optimal frequency (between 5 Hz to 20 Hz) in high-frequency rTMS of the left dorsolateral prefrontal cortex (DLPFC).

The basal ganglia area is the predilection site of stroke. The basal ganglia area, which is not an exact anatomical concept but one originating from neuroimaging, consists of the basal ganglia, the capsula interna and the surrounding white matter [6]; in other words, the basal ganglia area covers most of the subcortical structures that

For peer review only

constitute the limbic-cortical-striatal-pallidal-thalamic (LCSPT) circuit[7]. The limbic system is known to be closely related with emotional regulation[8], and as the LCSPT circuit is the most important part of the limbic system, LCSPT dysfunctions are thought to contribute to the occurrence of depression[9]. Investigators reported a high incidence of depression after cerebral infraction in the basal ganglia area[10]. Our group previously found that structural damage of the LCSPT circuit, as a result of white matter damage following cerebral infraction in the basal ganglia area, was associated with a high likeliness of PSD[11]. The nuclei in the LCSPT circuit are connected via nerve fibers, and damage of these nerve fibers disrupted the integrity of the white matter as shown by magnetic resonance imaging (MRI)[12].

As numerous studies have demonstrated the beneficial effect of rTMS treatment on white matter integrity[13-15], we hypothesized that the antidepressant effect of rTMS is related to its action on the LCSPT circuit in patients with depression following cerebral infraction in the basal ganglia area. The aim of this multicenter randomized controlled trial is to assess the effectiveness of rTMS in treating PSD and investigate the role of the LCSPT circuit in the therapeutic effect of rTMS for PSD.

METHODS

Study setting

The study will be conducted in two university hospitals, namely Zhujiang Hospital and Nanfang Hospital affiliated to Southern Medical University (Guangzhou, China) and in Sichuan Provincial Bayi Rehabilitation Hospital, a general hospital in Chengdu, China. The patients will be assessed for enrollment and eligible patients will be hospitalized for treatment in the departments of neurology in the 3 hospitals.

Trial design

The randomized, double-blind, placebo-controlled multicenter superiority trial is designed to recruit two parallel groups, and randomization will be performed with block randomization in a 1:1 allocation ratio. The study design is shown in the flow chart in Figure 1.

Participant enrollment and eligibility criteria

The participants will be recruited by advertisement on Internet and using posters in hospitals. The potential patients will be screened for enrollment according to the inclusion and exclusion criteria listed in **Table 1**.

Table 1 Inclusion and exclusion criteria

Criteria	Description
**Inclusion criteria**	1. First-time ischemic stroke with clinical and MRI or CT findings of basal ganglia ischemic stroke and a DSM-IV diagnosis of depression due to stroke (ICD-10-CM code 293.83[F06.32]);<br>2. Aged 25-75 years with a recent (from 3 weeks to 3 months) ischemic stroke;<br>3. Clear signs of neurological deficits in the acute phase;<br>4. Clear consciousness;<br>5. Right-handedness.
**Exclusion criteria**	1. Aphasia or severe cognitive impairment, severe hearing impairment,

For peer review only - http://bmjopen.bmj.com/site/about/guidelines.xhtml

or severe language comprehension deficits due to other causes;

2. Other cerebral diseases such as Parkinson's disease, encephalitis, dementia, multiple sclerosis, head injury, ect.;
3. Severe systemic disease or ongoing neoplasia;
4. Ongoing post-operative recovery;
5. Prior history of depressive disorders or major trauma within 1 year, severe depression or any other severe mental disorders;
6. Current or prior antidepressant use for any reason.
7. Addiction to drugs, alcohol or other substances;
8. Contraindications of MRI scan and rTMS treatment such as pacemaker implantation, a history of epilepsy, major head trauma, and seizures, ect;
9. Pregnant or breast-feeding women;
10. Participation in other clinical research projects;
11. Refusal to sign informed consent of this study.

Ethical considerations

This study has been approved by the Ethics Committee of Zhujiang Hospital of Southern Medical University. The methods, purpose and potential risks of this study will be fully explained to all the participants and their family members. Each patient will be asked to sign 3 informed consents before participating in this study.

Sample size calculation

The sample size was calculated based on the data of Valliengo et al[16], who reported a response rate of 0.041 in patients receiving sham rTMS treatment and a rate of 0.375 in patients having active rTMS treatment. Considering the low efficiency of sham rTMS treatment, we calculated the sample size using the following formula:

$$n_1 = n_2 = \frac{\left(Z_{\alpha/2} + Z_{\beta}\right)^2}{2\left(\sin^{-1}\sqrt{p_2} - \sin^{-1}\sqrt{p_1}\right)^2}。$$

The result showed that a sample size of 26 in each group was sufficient by assuming a two-tailed significance level of 5% with a statistical power of 90%. We estimate that the dropout rate will be less than 25%, and considering the need of randomisation, we assume that at least 33 participants are required in each group.

Randomisation and blinding

For randomisation of the patients, random numbers will be generated using SAS9.4 software by statisticians based on a randomized block design with a block length of 6. The magnetic stimulation coils for active and sham treatments have identical appearances and will be numbered by a statistician. The random numbers will be printed and sent to each trial center in sealed envelopes. Upon enrollment of an eligible patient, the investigator opens the envelope to reveal the number of the magnetic stimulation coil to be used, without knowing whether the patient receives active or sham treatment. In this double-blind trial, both the participants and investigators are blinded to the nature of treatment (active or sham).

Interventions

For peer review only

The participants enrolled in this trial will receive either active or sham rTMS treatment in addition to routine supportive treatments including antiplatelet agents, blood pressure control, neurotrophic therapy, physical therapy, and cognitive rehabilitation prescribed based on the condition of the individual participant and will not receive any other antidepressants, anxiolytics or hypnotics treatments that may have an impact on depression treatment during the study. All the participants will receive psychiatric and neuropsychological tests and MRI examination at the time of enrollment and at the end of treatment. To improve the participants' adherence to the intervention protocols, we will select the participants rigorously and cover the costs of neuropsychological assessment and MRI.

rTMS will be performed using a Mag Pro R30 device (Mag Venture Company, Frum, Denmark) via 121mm circular coils (MCF-125). The participants will receive active or sham treatment 5 days per week for 4 consecutive weeks. The motor threshold (MT) was defined as the lowest intensity required for motor cortex stimulation that causes a contraction of the abductor policies brevis muscle. The protocols of active rTMS treatment have been established by experts after discussions and the detailed parameters are listed in **Table 2**.

Table 2 Active rTMS treatment protocols

Localization	Frequency	Intensity	Times/ train	trains	Duration	Total times
Left DLPFC	10 Hz	110%MT	200	10	40 seconds	20
Right DLPFC	1 Hz	100%MT	30	10	10 seconds	20

Magnetic resonance imaging

Upon enrollment and at the end of this trial, the patient will receive MRI examination using a 3.0T PHILIPS Magnetic Trio Tim scanner. For the standard workup, MRI will be performed using the following scan parameters for different sequences: for T1-weighted sequence, time of echo (TE)=20 millisecond(ms), time of repetition (TR)=2000 ms, number of signal averaged (NSA)=1, slice=32, slice thickness=3 mm; for T2-weighted sequence, TE=80 ms, TR=3000 ms, NSA=2, slice=32, slice thickness=3 mm; for FLAIR sequence, TE=80 ms, TR=3000 ms, NSA=2, slice=32, slice thickness=3 mm; diffusion tensor imaging (DTI) using single short echo planar imaging (SE-EPI): TE=68 ms, TR=2300 ms, NSA=1, slice=32, slice thickness=3 mm, b_0=0, b_1=1000, matrix=512*512. Once the original data have been obtained, an experienced imaging physician will select the caudate nucleus, lentiform nucleus, dorsal medial nucleus of thalamus, and nucleus of the thalamus as the region of interests (ROIs) at the imaging layer of the basal ganglia. The three-dimensional fiber bundle imaging technique will be used to display the fibrous pathway of the dorsal basal ganglia system and the fibrous pathway of the ventral basal ganglia system, and the DTI parameters will be obtained including the fractional anisotropy (FA), apparent diffusion coefficient (ADC), neural fiber number (NFN). The MRI scanners at the 3 centres will be tested for homogeneity before the trial, while ROI selection and DTI

For peer review only - http://bmjopen.bmj.com/site/about/guidelines.xhtml

data acquisition will be performed by an experienced radiologist using PHILIP 2.1 software.

Outcome assessment

Baseline assessment

The demographic data will be collected from the patients, including age, gender, height, body weight, education level, occupation, and marital status. The vital signs will be measured including body temperature, pulse, respiration, and blood pressure. The lesion characteristics will be recorded including lateralization, number and size of the lesion. The data including the past history and treatment prescription, levels of blood lipids and blood glucose, liver function index and renal function index will be collected before randomisation by reviewing the medical records of the patients.

Primary efficacy parameters

The primary efficacy parameter is the change in HAMD-24 scores from the baseline at the end of the treatment. We plan to measure the response rate and remission rate as the primary efficacy parameters, determined by the changes in HAMD-24 scores from baseline at the end of the treatment. The response to treatment is defined as an at least 50% reduction of the HAMD-24 total score with a final HAMD-24 score below 9. Remission is defined as a reduction of the HAMD-24 total score by at least 50% from the baseline. As a valid and reliable scale for assessing the severity of depression, HAMD scale has been widely used in studies of PSD and shows a good sensitivity in assessing treatment responses. HAMD-24 scale consists of subscales that examine interest, anxiety, hopelessness, sleep status, and self-abasement, etc., and a higher score suggests a poorer condition of mental disorder[17]. HAMD-24 scale will be scored for 3 times at 0, 2, and 4 weeks after the commencement of treatment.

Secondary outcome measures

DTI results

Nerve fibers are important components of the LCSPT circuit apart from the cerebral cortex, corpus striatum and thalami. The nuclei are connected by abundant nerve fibers. DTI is a special sequence of MRI, which is currently the best non-invasive method to observe and track the nerve tracts. We will assess the DTI parameters of the patients including the fractional anisotropy (FA), apparent diffusion coefficient (ADC), neural fiber number (NFN) collected upon enrollment and at the end of the trial.

Neuropsychological tests

The neuropsychological status of the patients will be examined using the following scales or questionnaires: National Institutes of Health Stroke Scale (NIHSS), Activities of Daily Living Scale (ADLs), Montreal Cognitive Assessment (MoCA), which are widely used to evaluate neurological functions; Clinical Global Impressions scales(CGI), which is commonly used to measures of symptom severity and treatment response for patients with mental disorders; Aphasia Battery in Chinese (ABC), which is used to estimate the linguistic functions of Chinese patients; and Social Support Revalued Scale (SSRS) and Medical Coping Questionnaire score (MCMQ), which evaluate how social support influences emotions of the patient. All the tests will be performed at 0, 2 and 4 weeks after the commencement of the treatment.

For peer review only

Assessment of adverse events

All the adverse events (AEs) will be reported and recorded in the case report form (CRF) by investigators. The investigators will record the date of the occurrence, duration, and severity of the AEs, along with the treatments and consequences. Cases of serious AEs will be reported to the project leader and the ethics committee immediately, and the latter will evaluate whether the patient should receive further treatment. Those participants who suffer harm from this trial will be provided with compensation.

Quality control and data management

The trial protocol has been revised for several times by experts in neurology, rehabilitation, medical imaging, statistics and psychology. All the investigators have participated in the training sessions to ensure that they fully understand the trial protocols and the standard procedures before the study. Inspectors will be nominated to regularly visit each research center to monitor the implementation of the protocols and assess the compliance of the participants and investigators. All the data will be recorded on CRF by the investigators. A staff member will be appointed to manage all the CRFs and ensure the completeness of the data. The data will be double-entered by statisticians to ensure the accuracy of the data. Statisticians in our team will be responsible for data management and analysis.

Statistical analysis

The quantitative data, including the demographic indicators, laboratory test result, MRI results and neuropsychological test results, will be expressed as numbers, means with standard deviation or median with range or quartile range as appropriate. These data will be compared using independent-sample *t* test or Rank sum test depending on whether the data are normally distributed. The qualitative data, including the response and emission rates and incidences of the AEs, will be expressed as frequency or frequency rate and compared using χ^2 test between the two groups. All the data will be double-entered into Epidata software and analysed using SAS software by statisticians blinded to the participants' allocation. A *P* value less than 0.05 will be considered to indicate a statistically significant difference. Describe the exit reason, characteristics and last data collect, frequencies and percentage of missing data in each group and evaluate the imbalance by Chi-2 test or Fisher's exact test.

DISCUSSION

The conventional treatments of PSD include selective serotonin reuptake inhibitors (SSRIs)[18], tricyclic antidepressants (TCA)[19] and electroconvulsive therapy (ECT)[20], which are not always effective. Recent evidence has shown that rTMS has antidepressant actions [21, 22] and produces therapeutic effects comparable to those of ECT[23]. In spite of the various hypotheses that have been proposed, the exact mechanism underlying the antidepressant effect of rTMS remains unknown [24-26].

We determined the optimal frequency and intensity in rTMS treatment based on previous studies reporting positive antidepressant effect of the treatment[27-29], and chose the settings of pulses and sessions according to an evidence-based guideline[4]. As both high-frequency rTMS of the left DLPFC and low-frequency rTMS of the

For peer review only - http://bmjopen.bmj.com/site/about/guidelines.xhtml

right DLPFC are recommended in major depression treatment and considering that patients with PSD have more brain structural changes than those with primary depression, we chose bilateral rTMS of the DLPFC for treatment in this study.

White matter changes play important roles in the development of many neuropsychiatric diseases[30] including cognitive and emotional disorders[31]. As a non-invasive imaging technique, DTI provides an important modality for evaluating the white matter integrity in vivo, and the parameter FA can sensitively reflect the integrity of the white matter fibers while the ADC value allows sensitive detection of changes in the density and number of the nerve fibers[32]. Numerous studies have reported reduced FA values in the brain of patients with depression[33, 34], and these structural and functional brain abnormalities, particularly in the LCSPT system, are thought to be associated with the occurrence of depression [35]. Our previous study have confirmed that the occurrence of PSD was associated with neurological functional deficits following basal ganglia infarction, and the depression level was correlated with the reductions in the FA values of the bilateral pallidum and left putamen.

DTI offers an important means for evaluating the therapeutic effect in patients with PSD. Allendorfer et al compared the DTI data from 8 patients with post-stroke aphasia before and after a 10-day iTBS treatment and found significant increments in the FA values near the inferior and superior frontal gyri and the anterior corpus callosum[15]. Liao at al found significantly decreased white matter FA in the left middle frontal gyrus in patients with treatment-resistant depression (TRD) patients, and after 4 weeks of active rTMS treatment, the decreased FA values showed obvious improvement in the significant clusters [36]. Lyden et al also confirmed the positive effect of electroconvulsive therapy (ECT) in treatment of major depression by demonstrating significant increases in the FA values in the bilateral anterior cingulum and forceps minor after a one-week treatment[37]. Compared with major depression, PSD is associated with more severe structural and functional abnormalities of the brain, but currently researches evaluating white matter integrity in PSD patients by DTI remain insufficient. In this study, we will compare the DTI data before and after 4 weeks of rTMS treatment as a secondary outcome parameter to assess the effect of rTMS on white matter integrity in PSD patients.

This trial is expected to produce additional evidence to verify the effects of rTMS treatment on depression after ischemic stroke in the basal ganglia area and to explore the role of the LCSPT circuit in PSD. Findings from this study will fill the gaps in randomized controlled trials of rTMS for PSD in China, and contribute to the clinical evaluation of rTMS for PSD treatment and the improvement of the neural loop theory.

Contributions SZ, SP, MS and LZ participated in the conception and design of the trial. AC, JZ ,and LZ participated in planning the analysis of the data. YT, AC, LY ,participated in data collection, participants recruitment. AC, LY, SP, MS and LZ participated in the treatment of participants. YT and LZ participated in drafting the manuscript. All the authors discussed, revised and approved the final manuscript.

Acknowledgements Authors appreciate the effort of all people participating in this

trail.

Sponsor contact information Trial sponsor: Southern Medical University, Contact name: Mr. Yan Zhang, E-mail:zjyykyc@126.com.

Sponsor and funder this funding source had no role in the design of this study and will not have any role during its execution, analyses, interpretation of the data, or decision to submit results.

Funding This study was supported by grants of the Training Program of the Clinical Research Plan of Southern Medical University (LC2016PY031).

Competing interests None declared.

Patient consent Obtained Obtained.

Ethics approval The ethics committees of Zhujiang Hospital of Southern Medical University approved the study.

Provenance and peer review Not commissioned; peer reviewed for ethical and funding approval prior to submission.

Dissemination policy The results of this original research will be disseminated by publication in a peer-reviewed journal and presentation at international conferences.

Trial status The trial is currently ongoing in the stage of participant enrollment; the recruitment of patients started on 1 June, 2017 and will be completed in 31 May, 2020.

Open Access This is an Open Access article distributed in accordance with the terms of the Creative Commons Attribution (CC BY 4.0) license, which permits others to distribute, remix, adapt and build upon this work, for commercial use, provided the original works is properly cited. See: http://creativecommons.org/licenses/by/4.0/

Figure legends

Figure1： flow chart of the trial design.

REFERENCES

[1] Global, regional, and national life expectancy, all-cause mortality, and cause-specific mortality for 249 causes of death, 1980-2015: a systematic analysis for the Global Burden of Disease Study 2015. Lancet. 2016;**388**(10053):1459-1544 'doi': 10.1016/S0140-6736(16)31012-1['Copyright (c) 2016 The Author(s). Published by Elsevier Ltd. This is an Open Access article under the CC BY license. Published by Elsevier Ltd.. All rights reserved.:2016-10-08].

[2] Matsuzaki S, Hashimoto M, Yuki S, Koyama A, Hirata Y, Ikeda M. The relationship between post-stroke depression and physical recovery. J Affect Disord. 2015;**176**:56-60 'doi': 10.1016/j.jad.2015.01.020['Copyright (c) 2015 Elsevier B.V. All rights reserved.:2015-05-01].

[3] Mrazek DA, Hornberger JC, Altar CA, Degtiar I. A review of the clinical, economic, and societal burden of treatment-resistant depression: 1996-2013. Psychiatr Serv. 2014;**65**(8):977-987 'doi': 10.1176/appi.ps.201300059['2014-08-01].

[4] Lefaucheur JP, Andre-Obadia N, Antal A, Ayache SS, et al. Evidence-based guidelines on the therapeutic use of repetitive transcranial magnetic stimulation (rTMS). Clin Neurophysiol. 2014;**125**(11):2150-2206 'doi': 10.1016/j.clinph.2014.05.021['Copyright (c) 2014 International Federation of Clinical Neurophysiology.Published by Elsevier Ireland Ltd. All rights reserved.:2014-11-01].

[5] Shen X, Liu M, Cheng Y, Jia C, et al. Repetitive transcranial magnetic stimulation for the

treatment of post-stroke depression: A systematic review and meta-analysis of randomized controlled clinical trials. J Affect Disord. 2017;**211**:65-74 'doi': 10.1016/j.jad.2016.12.058['Copyright (c) 2017 Elsevier B.V. All rights reserved.:2017-03-15].

[6] Naritomi H, Sawada T, Kuriyama Y, Kinugawa H, Kaneko T, Takamiya M. Effect of chronic middle cerebral artery stenosis on the local cerebral hemodynamics. Stroke. 1985;**16**(2):214-2191985-03-01].

[7] Haber SN. The place of dopamine in the cortico-basal ganglia circuit. Neuroscience. 2014;**282**:248-257 'doi': 10.1016/j.neuroscience.2014.10.008['Copyright (c) 2014 IBRO. Published by Elsevier Ltd. All rights reserved.:2014-12-12].

[8] Joyce JN. The dopamine hypothesis of schizophrenia: limbic interactions with serotonin and norepinephrine. Psychopharmacology (Berl). 1993;**112**(1 Suppl):S16-S341993-01-19].

[9] Berton O, Nestler EJ. New approaches to antidepressant drug discovery: beyond monoamines. Nat Rev Neurosci. 2006;**7**(2):137-151 'doi': 10.1038/nrn1846['2006-02-01].

[10] Wu RH, Li Q, Tan Y, Liu XY, Huang J. Depression in silent lacunar infarction: a cross-sectional study of its association with location of silent lacunar infarction and vascular risk factors. Neurol Sci. 2014;**35**(10):1553-1559 'doi': 10.1007/s10072-014-1794-5['2014-10-01].

[11] Jia-shan TU, Zhen-hua L, Fan-heng H, Ai-min C, et al. Evaluation of depression status following basal ganglia infarction by diffusion tensor magnetic resonance imaging. Chinese Journal of Neurology. 2012;**45**(8):590-594 'doi': 10.3760/cma.j.issn.1006-7876.2012.08.010['.

[12] Nugent AC, Davis RM, Zarate CJ, Drevets WC. Reduced thalamic volumes in major depressive disorder. Psychiatry Res. 2013;**213**(3):179-185 'doi': 10.1016/j.pscychresns.2013.05.004['Published by Elsevier Ireland Ltd.:2013-09-30].

[13] Kozel FA, Johnson KA, Nahas Z, Nakonezny PA, et al. Fractional anisotropy changes after several weeks of daily left high-frequency repetitive transcranial magnetic stimulation of the prefrontal cortex to treat major depression. J ECT. 2011;**27**(1):5-10 'doi': 10.1097/YCT.0b013e3181e6317d['2011-03-01].

[14] Yang W, Liu TT, Song XB, Zhang Y, et al. Comparison of different stimulation parameters of repetitive transcranial magnetic stimulation for unilateral spatial neglect in stroke patients. J Neurol Sci. 2015;**359**(1-2):219-225 'doi': 10.1016/j.jns.2015.08.1541['Copyright (c) 2015. Published by Elsevier B.V.:2015-12-15].

[15] Allendorfer JB, Storrs JM, Szaflarski JP. Changes in white matter integrity follow excitatory rTMS treatment of post-stroke aphasia. Restor Neurol Neurosci. 2012;**30**(2):103-113 'doi': 10.3233/RNN-2011-0627['2012-01-20].

[16] Valiengo LC, Goulart AC, de Oliveira JF, Bensenor IM, Lotufo PA, Brunoni AR. Transcranial direct current stimulation for the treatment of post-stroke depression: results from a randomised, sham-controlled, double-blinded trial. J Neurol Neurosurg Psychiatry. 2016 'doi': 10.1136/jnnp-2016-314075['Published by the BMJ Publishing Group Limited. For permission to use (where not already granted under a licence) please go to http://www.bmj.com/company/products-services/rights-and-licensing/.:2016-11-04].

[17] Williams JB, Kobak KA, Bech P, Engelhardt N, et al. The GRID-HAMD: standardization of the Hamilton Depression Rating Scale. Int Clin Psychopharmacol. 2008;**23**(3):120-129 'doi': 10.1097/YIC.0b013e3282f948f5['2008-05-01].

[18] Mead GE, Hsieh CF, Hackett M. Selective serotonin reuptake inhibitors for stroke recovery. JAMA. 2013;**310**(10):1066-1067 'doi': 10.1001/jama.2013.107828['2013-09-11].

For peer review only

[19] Capaldi VN, Wynn GH. Post stroke depression: treatments and complications in a young adult. Psychiatr Q. 2010;**81**(1):73-79 'doi': 10.1007/s11126-009-9120-8['2010-03-01].

[20] Berggren A, Gustafson L, Hoglund P, Johanson A. A long-term follow-up of clinical response and regional cerebral blood flow changes in depressed patients treated with ECT. J Affect Disord. 2014;**167**:235-243 'doi': 10.1016/j.jad.2014.06.005['Copyright (c) 2014 The Authors. Published by Elsevier B.V. All rights reserved.:2014-01-20].

[21] Kim BR, Kim DY, Chun MH, Yi JH, Kwon JS. Effect of repetitive transcranial magnetic stimulation on cognition and mood in stroke patients: a double-blind, sham-controlled trial. Am J Phys Med Rehabil. 2010;**89**(5):362-368 'doi': 10.1097/PHM.0b013e3181d8a5b1['2010-05-01].

[22] Levkovitz Y, Isserles M, Padberg F, Lisanby SH, et al. Efficacy and safety of deep transcranial magnetic stimulation for major depression: a prospective multicenter randomized controlled trial. World Psychiatry. 2015;**14**(1):64-73 'doi': 10.1002/wps.20199['(c) 2015 World Psychiatric Association.:2015-02-01].

[23] Ren J, Li H, Palaniyappan L, Liu H, et al. Repetitive transcranial magnetic stimulation versus electroconvulsive therapy for major depression: a systematic review and meta-analysis. Prog Neuropsychopharmacol Biol Psychiatry. 2014;**51**:181-189 'doi': 10.1016/j.pnpbp.2014.02.004['Copyright (c) 2014 Elsevier Inc. All rights reserved.:2014-06-03].

[24] Yulug B, Hanoglu L, Tavli AM, Yilmaz NH, Kilic E. The Brain Protective Effect of rTMS (Repetitive Transcranial Magnetic Stimulation) in Depression: A Mini-Review in Animal Studies. Med Chem. 2016;**12**(6):500-5052016-01-20].

[25] Wang HN, Wang L, Zhang RG, Chen YC, et al. Anti-depressive mechanism of repetitive transcranial magnetic stimulation in rat: the role of the endocannabinoid system. J Psychiatr Res. 2014;**51**:79-87 'doi': 10.1016/j.jpsychires.2014.01.004['Copyright (c) 2014 Elsevier Ltd. All rights reserved.:2014-04-01].

[26] Peng H, Zheng H, Li L, Liu J, et al. High-frequency rTMS treatment increases white matter FA in the left middle frontal gyrus in young patients with treatment-resistant depression. J Affect Disord. 2012;**136**(3):249-257 'doi': 10.1016/j.jad.2011.12.006['Copyright (c) 2011 Elsevier B.V. All rights reserved.:2012-02-01].

[27] Jorge RE, Robinson RG, Tateno A, Narushima K, et al. Repetitive transcranial magnetic stimulation as treatment of poststroke depression: a preliminary study. Biol Psychiatry. 2004;**55**(4):398-405 'doi': 10.1016/j.biopsych.2003.08.017['2004-02-15].

[28] Herbsman T, Avery D, Ramsey D, Holtzheimer P, et al. More lateral and anterior prefrontal coil location is associated with better repetitive transcranial magnetic stimulation antidepressant response. Biol Psychiatry. 2009;**66**(5):509-515 'doi': 10.1016/j.biopsych.2009.04.034['2009-09-01].

[29] Avery DH, Holtzheimer PR, Fawaz W, Russo J, et al. A controlled study of repetitive transcranial magnetic stimulation in medication-resistant major depression. Biol Psychiatry. 2006;**59**(2):187-194 'doi': 10.1016/j.biopsych.2005.07.003['2006-01-15].

[30] Filley CM. The behavioral neurology of cerebral white matter. Neurology. 1998;**50**(6):1535-15401998-06-01].

[31] Filley CM, Fields RD. White matter and cognition: making the connection. J Neurophysiol. 2016;**116**(5):2093-2104 'doi': 10.1152/jn.00221.2016['2016-11-01].

[32] Peng H, Zheng H, Li L, Liu J, et al. High-frequency rTMS treatment increases white matter FA in the left middle frontal gyrus in young patients with treatment-resistant depression. J Affect Disord. 2012;**136**(3):249-257 'doi': 10.1016/j.jad.2011.12.006['Copyright (c) 2011 Elsevier B.V. All rights

For peer review only

reserved.:2012-02-01].

[33] Kozel FA, Johnson KA, Nahas Z, Nakonezny PA, et al. Fractional anisotropy changes after several weeks of daily left high-frequency repetitive transcranial magnetic stimulation of the prefrontal cortex to treat major depression. J ECT. 2011;**27**(1):5-10 'doi': 10.1097/YCT.0b013e3181e6317d['2011-03-01].

[34] Peng H, Zheng H, Li L, Liu J, et al. High-frequency rTMS treatment increases white matter FA in the left middle frontal gyrus in young patients with treatment-resistant depression. J Affect Disord. 2012;**136**(3):249-257 'doi': 10.1016/j.jad.2011.12.006['Copyright (c) 2011 Elsevier B.V. All rights reserved.:2012-02-01].

[35] Sheline YI. Neuroimaging studies of mood disorder effects on the brain. Biol Psychiatry. 2003;**54**(3):338-3522003-08-01].

[36] Liao Y, Tang J, Ma M, Wu Z, et al. Frontal white matter abnormalities following chronic ketamine use: a diffusion tensor imaging study. Brain. 2010;**133**(Pt 7):2115-2122 'doi': 10.1093/brain/awq131['2010-07-01].

[37] Lyden H, Espinoza RT, Pirnia T, Clark K, et al. Electroconvulsive therapy mediates neuroplasticity of white matter microstructure in major depression. Transl Psychiatry. 2014;**4**:e380 'doi': 10.1038/tp.2014.21['2014-04-08].

For peer review only

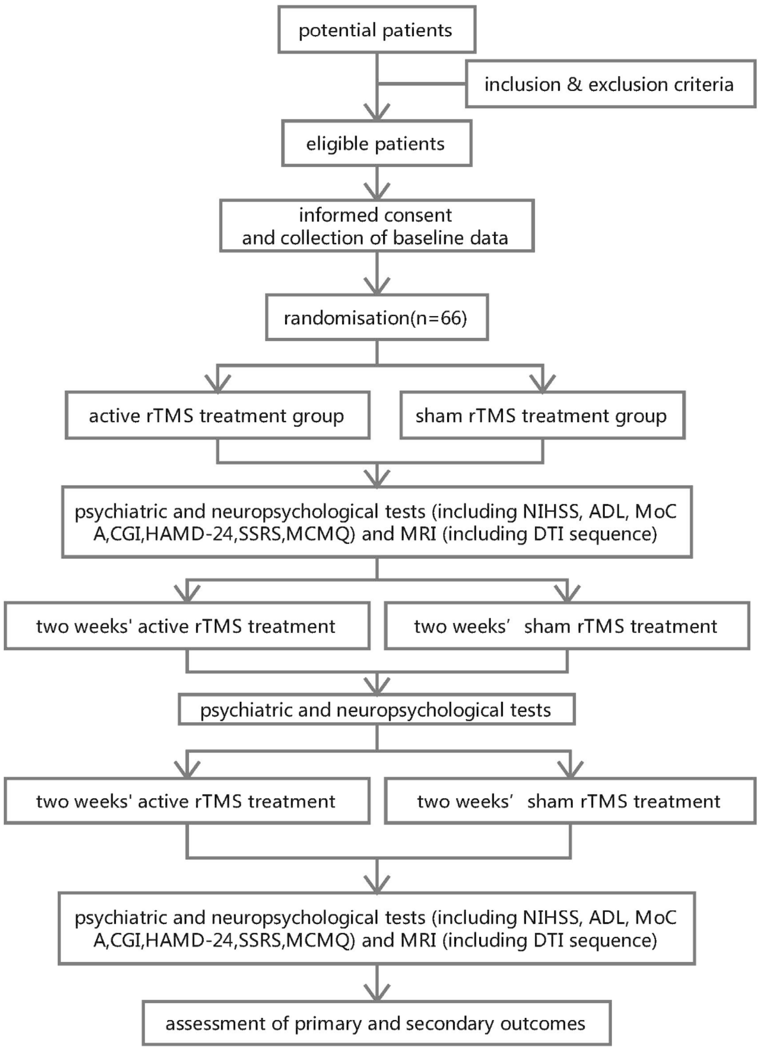

Figure1: Flow chart of the study design

173x228mm (300 x 300 DPI)

SPIRIT
STANDARD PROTOCOL ITEMS: RECOMMENDATIONS FOR INTERVENTIONAL TRIALS

SPIRIT 2013 Checklist: Recommended items to address in a clinical trial protocol and related documents*

Section/item	ItemNo	Description	Description
**Administrative information**			
Title	1	Descriptive title identifying the study design, population, interventions, and, if applicable, trial acronym	Repetitive transcranial magnetic stimulation for depression after basal ganglia ischemic stroke: protocol for a multicenter, randomized, double-blind, placebo-controlled trial
Trial registration	2a	Trial identifier and registry name. If not yet registered, name of intended registry	ClinicalTrials.gov, Identifier: NCT03159351
	2b	All items from the World Health Organization Trial Registration Data Set	
Protocol version	3	Date and version identifier	19/02/2017
Funding	4	Sources and types of financial, material, and other support	Training Program of the Clinical Research Plan of Southern Medical University (LC2016PY031).c
Roles and responsibilities	5a	Names, affiliations, and roles of protocol contributors	SZ, SP, MS and LZ participated in the conception and design of the trial. AC, JZ ,and LZ participated in planning the analysis of the data. YT, AC, LY ,participated in data collection, participants recruitment. AC, LY, SP, MS and LZ participated in the treatment of participants. YT and LZ participated in drafting the manuscript. All the authors discussed, revised and approved the final manuscript.
	5b	Name and contact information for the trial sponsor	Yan Zhang, the administrator of the Clinical Research Plan of Southern Medical University，E-mail: zjyykyc@126.com.

For peer review only - http://bmjopen.bmj.com/site/about/guidelines.xhtml

5c	Role of study sponsor and funders, if any, in study design; collection, management, analysis, and interpretation of data; writing of the report; and the decision to submit the report for publication, including whether they will have ultimate authority over any of these activities	This funding source had no role in the design of this study and will not have any role during its execution, analyses, interpretation of the data, or decision to submit results.
5d	Composition, roles, and responsibilities of the coordinating centre, steering committee, endpoint adjudication committee, data management team, and other individuals or groups overseeing the trial, if applicable (see Item 21a for data monitoring committee)	NA

Introduction

For peer review only - http://bmjopen.bmj.com/site/about/guidelines.xhtml

For peer review only

Background and rationale	6a	Description of research question and justification for undertaking the trial, including summary of relevant studies (published and unpublished) examining benefits and harms for each intervention	Stroke is a global health problem and in 2015, the number of deaths related with stroke reached 6.326 million around the world, among which 45.89% were attributed to ischemic stroke Depression occurs frequently in the stroke survivors and has a significant impact on post-stroke rehabilitation. The conventional treatment of post-stroke depression (PSD) relies on supportive therapy and medications, but a proportion of patients can remain treatment-refractory. Studies has shown that repetitive transcranial magnetic stimulation (rTMS) has a definite antidepressant effect on major depression, and a recent meta-analysis also suggests the beneficial effects of rTMS on PSD, but such studies should be carefully examined for their heterogeneity and potential biases. To our knowledge, currently no studies have been reported to examine the effect of rTMS on PSD in China, and the mechanism by which rTMS improves PSD remains to be clarified. In addition, the stimulation parameter settings in rTMS treatment still awaits standardization, and it is difficult to determine the optimal frequency (between 5 Hz to 20 Hz) in high-frequency rTMS of the left dorsolateral prefrontal cortex (DLPFC). The basal ganglia area is the predilection site of stroke. The basal ganglia area, which is not an exact anatomical concept but one originating from neuroimaging, consists of the basal ganglia, the capsula interna and the surrounding white matter ; in other words, the basal ganglia area covers most of the subcortical structures that constitute the limbic-cortical-striatal-pallidal-thalamic (LCSPT) circuit. The limbic system is known to be closely related with emotional regulation, and as the LCSPT circuit is the most important part of the limbic system, LCSPT dysfunctions are thought to contribute to the occurrence of depression. Investigators reported a high incidence of depression after cerebral infraction in the basal ganglia area. Our group previously found that structural damage of the LCSPT circuit, as a result of white matter damage following cerebral infraction in the basal ganglia area, was associated with a high likeliness of PSD. The nuclei in the LCSPT circuit are connected via nerve fibers, and damage of these nerve fibers disrupted the integrity of the white matter as shown by magnetic resonance imaging (MRI).

For peer review only - http://bmjopen.bmj.com/site/about/guidelines.xhtml

	6b	Explanation for choice of comparators	To our knowledge, currently no studies have been reported to examine the effect of rTMS on PSD in China, and the mechanism by which rTMS improves PSD remains to be clarified. In addition, the stimulation parameter settings in rTMS treatment still awaits standardization, and it is difficult to determine the optimal frequency (between 5 Hz to 20 Hz) in high-frequency rTMS of the left dorsolateral prefrontal cortex (DLPFC). We determined the optimal frequency and intensity in rTMS treatment based on previous studies reporting positive antidepressant effect of the treatment, and chose the settings of pulses and sessions according to an evidence-based guideline. As both high-frequency rTMS of the left DLPFC and low-frequency rTMS of the right DLPFC are recommended in major depression treatment and considering that patients with PSD have more brain structural changes than those with primary depression, we chose bilateral rTMS of the DLPFC for treatment in this study.
Objectives	7	Specific objectives or hypotheses	We hypothesized that the antidepressant effect of rTMS is related to its action on the LCSPT circuit in patients with depression following cerebral infraction in the basal ganglia area. The aim of this multicenter randomized controlled trial is to assess the effectiveness of rTMS in treating PSD and investigate the role of the LCSPT circuit in the therapeutic effect of rTMS for PSD.
Trial design	8	Description of trial design including type of trial (eg, parallel group, crossover, factorial, single group), allocation ratio, and framework (eg, superiority, equivalence, noninferiority, exploratory)	The trial is designed as a superiority randomized, double-blind, placebo-controlled multicenter with two parallel groups and randomization will be performed as block randomization with a 1:1 allocation.
**Methods: Participants, interventions, and outcomes**			
Study setting	9	Description of study settings (eg, community clinic, academic hospital) and list of countries where data will be collected. Reference to where list of study sites can be obtained	The study will be conducted in two university hospitals, namely Zhujiang Hospital and Nanfang Hospital affiliated to Southern Medical University (Guangzhou, China) and in Sichuan Provincial Bayi Rehabilitation Hospital, a general hospital in Chengdu, China.

For peer review only - http://bmjopen.bmj.com/site/about/guidelines.xhtml

Eligibility criteria	10	Inclusion and exclusion criteria for participants. If applicable, eligibility criteria for study centres and individuals who will perform the interventions (eg, surgeons, psychotherapists)	Inclusion criteria:1. First-time ischemic stroke with clinical and MRI or CT findings of basal ganglia ischemic stroke and a DSM-IV diagnosis of depression due to stroke (ICD-10-CM code 293.83[F06.32]); 2. Aged 25-75 years with a recent (from 3 weeks to 3 months) ischemic stroke; 3. Clear signs of neurological deficits in the acute phase;4. Clear consciousness;5. Right-handedness.<br>Exclusion criteria:1.Aphasia or severe cognitive impairment, severe hearing impairment, or severe language comprehension deficits due to other causes; 2.Other cerebral diseases such as Parkinson's disease, encephalitis, dementia, multiple sclerosis, head injury, ect.; 3.Severe systemic disease or ongoing neoplasia; 4.Ongoing post-operative recovery; 5.Prior history of depressive disorders or major trauma within 1 year, severe depression or any other severe mental disorders; 6.Current or prior antidepressant use for any reason. 7.Addiction to drugs, alcohol or other substances; 8.Contraindications of MRI scan and rTMS treatment such as pacemaker implantation, a history of epilepsy, major head trauma, and seizures, ect; 9.Pregnant or breast-feeding women; 10.Participation in other clinical research projects; 11.Refusal to sign informed consent of this study.
Interventions	11a	Interventions for each group with sufficient dcetail to allow replication, including how and when they will be administered	rTMS will be performed using a Mag Pro R30 device (Mag Venture Company, Frum, Denmark) via 121mm circular coils (MCF-125). The participants will receive active or sham treatment 5 days per week for 4 consecutive weeks. The motor threshold (MT) was defined as the lowest intensity required for motor cortex stimulation that causes a contraction of the abductor policies brevis muscle. The protocols of active rTMS treatment have been established by experts after discussions and the detailed parameters are listed in Table 2.
	11b	Criteria for discontinuing or modifying allocated interventions for a given trial participant (eg, drug dose change in response to harms, participant request, or improving/worsening disease)	We will discontinue the allocated interventions once the disease has significant worsening.

For peer review only - http://bmjopen.bmj.com/site/about/guidelines.xhtml

	11c	Strategies to improve adherence to intervention protocols, and any procedures for monitoring adherence (eg, drug tablet return, laboratory tests)	To improve adherence to intervention protocols, we will select the participants rigorously and compensate the cost of neuropsychological assessment and MRI.
	11d	Relevant concomitant care and interventions that are permitted or prohibited during the trial	The participants enrolled in this trial will receive either active or sham rTMS treatment in addition to routine supportive treatments including antiplatelet agents, blood pressure control, neurotrophic therapy, physical therapy, and cognitive rehabilitation prescribed based on the condition of the individual participant and will not receive any other antidepressants, anxiolytics or hypnotics treatments that may have an impact on depression treatment during the study. All the participants will receive psychiatric and neuropsychological tests and MRI examination at the time of enrollment and at the end of treatment.
Outcomes	12	Primary, secondary, and other outcomes, including the specific measurement variable (eg, systolic blood pressure), analysis metric (eg, change from baseline, final value, time to event), method of aggregation (eg, median, proportion), and time point for each outcome. Explanation of the clinical relevance of chosen efficacy and harm outcomes is strongly recommended	The primary outcome is the measurement of 24-item Hamilton Depression Rating Scale (HAMD-24) scores, and the secondary outcomes include diffusion tensor imaging (DTI) results and the results of neuropsychological tests including National Institutes of Health Stroke Scale (NIHSS), Activities of Daily Living Scale (ADLs), Montreal Cognitive Assessment (MoCA), Clinical Global Impressions scales(CGI), Aphsia Battery in Chinese (ABC), Social Support Revalued Scale (SSRS) and Medical Coping Questionnaire (MCMQ).
Participant timeline	13	Time schedule of enrolment, interventions (including any run-ins and washouts), assessments, and visits for participants. A schematic diagram is highly recommended (see Figure)	See Figure 1.

For peer review only - http://bmjopen.bmj.com/site/about/guidelines.xhtml

Sample size	14	Estimated number of participants needed to achieve study objectives and how it was determined, including clinical and statistical assumptions supporting any sample size calculations	The primary efficacy parameter is the change in HAMD-24 scores from the baseline to the end of the treatment. The sample size was calculated based on the data of Valliengo et al[19], who reported a response rate of 0.041 in patients receiving sham rTMS treatment and a rate of 0.375 in patients having active rTMS treatment. Considering the low efficiency of sham rTMS treatment, we calculated the sample size using the following formula: $$n_1 = n_2 = \frac{(Z_{\alpha/2} + Z_\beta)^2}{2(\sin^{-1}\sqrt{p_2} - \sin^{-1}\sqrt{p_1})^2} 。$$ The result showed that a sample size of 26 in each group was sufficient by assuming a two-tailed significance level of 5% and a statistical power of 90%. We estimate that the dropout rate will be less than 25%, and considering the need of randomisation, we assume that at least 33 participants are required in each group.
Recruitment	15	Strategies for achieving adequate participant enrolment to reach target sample size	The participants will be recruited by advertisement on Internet and using posters in hospitals.

Methods: Assignment of interventions (for controlled trials)

Allocation:

Sequence generation	16a	Method of generating the allocation sequence (eg, computer-generated random numbers), and list of any factors for stratification. To reduce predictability of a random sequence, details of any planned restriction (eg, blocking) should be provided in a separate document that is unavailable to those who enrol participants or assign interventions	Random numbers will be generated using SAS9.4 software by statisticians based on a randomized block design with a block length of 6.

For peer review only - http://bmjopen.bmj.com/site/about/guidelines.xhtml

Allocation concealment mechanism	16b	Mechanism of implementing the allocation sequence (eg, central telephone; sequentially numbered, opaque, sealed envelopes), describing any steps to conceal the sequence until interventions are assigned	The magnetic stimulation coils for active and sham treatments have identical appearances and will be numbered by a statistician. The random numbers will be printed and sent to each trial center in sealed envelopes. Upon enrollment of an eligible patient, the investigator opens the envelope to receive the number of the magnetic stimulation coil to be used, without knowing whether the patient receives active or sham treatment.
Implementation	16c	Who will generate the allocation sequence, who will enrol participants, and who will assign participants to interventions	The project leader will generate the allocation sequence, investigators will enroll participants, and the assignation of participants to interventions based on computerized randomisation.
Blinding (masking)	17a	Who will be blinded after assignment to interventions (eg, trial participants, care providers, outcome assessors, data analysts), and how	In this double-blind trial, both the participants and investigators are blinded to the nature of treatment (active or sham).
	17b	If blinded, circumstances under which unblinding is permissible, and procedure for revealing a participant's allocated intervention during the trial	We will discontinue the allocated interventions once the disease has significant worsening.

Methods: Data collection, management, and analysis

w only

Data collection methods	18a	Plans for assessment and collection of outcome, baseline, and other trial data, including any related processes to promote data quality (eg, duplicate measurements, training of assessors) and a description of study instruments (eg, questionnaires, laboratory tests) along with their reliability and validity, if known. Reference to where data collection forms can be found, if not in the protocol	Baseline assessment<br>The demographic data will be collected from the patients, including age, gender, height, body weight, education level, occupation, and marital status. The vital signs will be measured including body temperature, pulse, respiration, and blood pressure. The lesion characteristics including the lateralization, number and size of the lesion.The data including the past history and treatment prescription, levels of blood lipids and blood glucose, liver function index and renal function index will be collected before randomisation by reviewing the medical records of the patients.<br>We plan to measure the response rate and remission rate as primary efficacy parameters, namely the changes in HAMD-24 scores from baseline to the end of the treatment. HAMD-24 scale will be scored for 3 times at 0, 2, and 4 weeks after the commencement of treatment.<br>DTI parameters and Neuropsychological tests are Secondary outcome measures. DTI parameters including the fractional anisotropy (FA), apparent diffusion coefficient (ADC), neural fiber number (NFN) will be scored at 0, and 4 weeks after the commencement of treatment.<br>neuropsychological tests including National Institutes of Health Stroke Scale (NIHSS), Activities of Daily Living Scale (ADLs), Montreal Cognitive Assessment (MoCA), Clinical Global Impressions scales(CGI), Aphsia Battery in Chinese (ABC), Social Support Revalued Scale (SSRS) and Medical Coping Questionnaire (MCMQ) will be implemented at 0, 2 and 4 weeks after the commencement of the treatment.
	18b	Plans to promote participant retention and complete follow-up, including list of any outcome data to be collected for participants who discontinue or deviate from intervention protocols	The methods, purpose and potential risks of this study will be fully explained to all the participants and their family members. Each patient will be asked to sign 3 informed consents before participating in this study.At the end of the patient's treatment, the patient was informed about the follow-up, and a telephone call will be made in the fourth week after the end-point to remind them follow-up.

For peer review only - http://bmjopen.bmj.com/site/about/guidelines.xhtml

Data management	19	Plans for data entry, coding, security, and storage, including any related processes to promote data quality (eg, double data entry; range checks for data values). Reference to where details of data management procedures can be found, if not in the protocol	The trial protocol has been revised for several times by experts in neurology, rehabilitation, medical imaging, statistics and psychology. All the investigators have participated in the training sessions to ensure that they fully understand the trial protocols and the standard procedures before the study. Inspectors will be nominated to regularly visit each research center to monitor the implementation of the protocols and assess the compliance of the participants and investigators. All the data will be recorded on CRF by the investigators. A staff member will be appointed to manage all the CRFs and ensure the completeness of the data. The data will be double-entered by statisticians to ensure the accuracy of the data. Statisticians in our team will be responsible for data management and analysis.
Statistical methods	20a	Statistical methods for analysing primary and secondary outcomes. Reference to where other details of the statistical analysis plan can be found, if not in the protocol	The quantitative data, including the demographic indicators, laboratory test result, MRI results and neuropsychological test results, will be expressed as numbers, means with standard deviation or median with range or quartile range as appropriate. These data will be compared using independent-sample *t* test or Rank sum test depending on whether the data are normally distributed. The qualitative data, including the response and emission rates and incidences of the AEs, will be expressed as frequency or frequency rate and compared using χ^2 test between the two groups. All the data will be double-entered into Epidata software and analysed using SAS software by statisticians blinded to the participants' allocation. A *P* value less than 0.05 will be considered to indicate a statistically significant difference.
	20b	Methods for any additional analyses (eg, subgroup and adjusted analyses)	NA
	20c	Definition of analysis population relating to protocol non-adherence (eg, as randomised analysis), and any statistical methods to handle missing data (eg, multiple imputation)	Missing data will be described in terms of exit data, exit reason, treatment arm, frequencies and percentage of missing data in each group. The data imbalances will be evaluated by Chi-2 test of Fisher's exact test.

Methods: Monitoring

For peer review only - http://bmjopen.bmj.com/site/about/guidelines.xhtml

Data monitoring	21a	Composition of data monitoring committee (DMC); summary of its role and reporting structure; statement of whether it is independent from the sponsor and competing interests; and reference to where further details about its charter can be found, if not in the protocol. Alternatively, an explanation of why a DMC is not needed	An Internal independent data monitoring is planned. Data managers only get the data by Case Report Form (CRF) for data management, does not participate in the clinical treatment and psychological assessment.
	21b	Description of any interim analyses and stopping guidelines, including who will have access to these interim results and make the final decision to terminate the trial	No interim analyses is envisaged.
Harms	22	Plans for collecting, assessing, reporting, and managing solicited and spontaneously reported adverse events and other unintended effects of trial interventions or trial conduct	All adverse effect are reported in the CRF.
Auditing	23	Frequency and procedures for auditing trial conduct, if any, and whether the process will be independent from investigators and the sponsor	An annual audit by the Delegation of Research Department of Southern Medical University is provided.
**Ethics and dissemination**			
Research ethics approval	24	Plans for seeking research ethics committee/institutional review board (REC/IRB) approval	The ethics committees of Zhujiang Hospital of Southern Medical University approved the study(Identifier: 2016-SJNK-005)

For peer review only - http://bmjopen.bmj.com/site/about/guidelines.xhtml

Protocol amendments	25	Plans for communicating important protocol modifications (eg, changes to eligibility criteria, outcomes, analyses) to relevant parties (eg, investigators, REC/IRBs, trial participants, trial registries, journals, regulators)	NA
Consent or assent	26a	Who will obtain informed consent or assent from potential trial participants or authorised surrogates, and how (see Item 32)	The methods, purpose and potential risks of this study will be fully explained to all the participants and their family members. Each patient will be asked to sign 3 informed consents before participating in this study.
	26b	Additional consent provisions for collection and use of participant data and biological specimens in ancillary studies, if applicable	NA
Confidentiality	27	How personal information about potential and enrolled participants will be collected, shared, and maintained in order to protect confidentiality before, during, and after the trial	Personal information about potential and enrolled participants will be either stored in the medical record or in research record and identified with initials and number of inclusion. Records are stored securely in locked areas.
Declaration of interests	28	Financial and other competing interests for principal investigators for the overall trial and each study site	Authors have no conflicts of interest to declare.
Access to data	29	Statement of who will have access to the final trial dataset, and disclosure of contractual agreements that limit such access for investigators	Data-manager, study statistician, investigators, will have access to the final trial dataset.
Ancillary and post-trial care	30	Provisions, if any, for ancillary and post-trial care, and for compensation to those who suffer harm from trial participation	A compensation of the cost of neuropsychological assessment and MRI.

For peer review only - http://bmjopen.bmj.com/site/about/guidelines.xhtml

Dissemination policy	31a	Plans for investigators and sponsor to communicate trial results to participants, healthcare professionals, the public, and other relevant groups (eg, via publication, reporting in results databases, or other data sharing arrangements), including any publication restrictions	The results of this original research will be disseminated by publication in a peer-reviewed journal and presentation at international conferences.
	31b	Authorship eligibility guidelines and any intended use of professional writers	NA
	31c	Plans, if any, for granting public access to the full protocol, participant-level dataset, and statistical code	NA
**Appendices**			
Informed consent materials	32	Model consent form and other related documentation given to participants and authorised surrogates	Consent form has been validated by the ethical committee.
Biological specimens	33	Plans for collection, laboratory evaluation, and storage of biological specimens for genetic or molecular analysis in the current trial and for future use in ancillary studies, if applicable	NA

*It is strongly recommended that this checklist be read in conjunction with the SPIRIT 2013 Explanation & Elaboration for important clarification on the items. Amendments to the protocol should be tracked and dated. The SPIRIT checklist is copyrighted by the SPIRIT Group under the Creative Commons "Attribution-NonCommercial-NoDerivs 3.0 Unported" license.

For peer review only

BMJ Open

Repetitive transcranial magnetic stimulation for depression after basal ganglia ischemic stroke: protocol for a multicenter, randomized, double-blind, placebo-controlled trial

Journal:	*BMJ Open*
Manuscript ID	bmjopen-2017-018011.R2
Article Type:	Protocol
Date Submitted by the Author:	09-Nov-2017
Complete List of Authors:	Tang, Ying; Zhujiang Hospital of Southern Medical University,<br>Chen, Aimin; Zhujiang Hospital of Southern Medical University<br>Zhu, Shuzhen; Zhujiang Hospital of Southern Medical University<br>Yang, Li; Zhujiang Hospital of Southern Medical University<br>Zhou, Jiyuan; Southern Medical University, Department of Biostatistics, School of Public Health<br>Pan, Suyue; Southern Medical University Nanfang Hospital<br>Shao, Ming; Sichuan Bayi Rehabilitation Center<br>Zhao, Lianxu; Zhujiang Hospital of Southern Medical University
**Primary Subject Heading**:	Neurology
Secondary Subject Heading:	Mental health
Keywords:	repetitive transcranial magnetic stimulation, post-stroke depression, ischemic stroke

SCHOLARONE™
Manuscripts

For peer review only

Repetitive transcranial magnetic stimulation for depression after basal ganglia ischemic stroke: protocol for a multicenter, randomized, double-blind, placebo-controlled trial

Ying Tang[1], Aimin Chen[2], Shuzhen Zhu[3], Li Yang[4], Jiyuan Zhou[5], Suyue Pan[6], Min Shao[7], Lianxu Zhao[8*]

Author information:

1 Zhujiang Hospital of Southern Medical University, Guangzhou, Guangdong, China. E-mail: tangying05@qq.com.

2 Department of Neurology, Zhujiang Hospital of Southern Medical University, Guangzhou, Guangdong, China. E-mail: doctoramchen@163.com.

3 Department of Neurology, Zhujiang Hospital of Southern Medical University, Guangzhou, Guangdong, China. E-mail: miraclezsz@qq.com.

4 Department of Neurology, Zhujiang Hospital of Southern Medical University, Guangzhou, Guangdong, China. E-mail: ylsnn09@163.com.

5 School of Public Health, Southern Medical University, Guangzhou, Guangdong, China. E-mail: zhoujiyuan5460@hotmail.com.

6 Department of Neurology, Nanfang Hospital of Southern Medical University, Guangzhou, Guangdong, China. E-mail: pansuyue82@qq.com.

7 Sichuan Bayi Rehabilitation Center, Chengdu, Sichuan, China. E-mail: 1961523453@qq.com.

8 Department of Neurology, Zhujiang Hospital of Southern Medical University, Guangzhou, Guangdong, China. E-mail: zhaolianxu@smu.edu.cn.

*Corresponding author, postal address: No.253, Industrial Avenue, Zhujiang Hospital of Southern Medical University, Guangzhou, Guangdong, China, 510280, E-mail: zhaolianxu@smu.edu.cn, phone: +86 2062783082.

Keywords: repetitive transcranial magnetic stimulation, post-stroke depression, ischemic stroke.

Word count: 3529 words.

ABSTRACT

Introduction: Studies suggest that repetitive transcranial magnetic stimulation (rTMS) is effective for treatment of depression and promotes the repair of white matter. This study aims to assess the effectiveness of rTMS in treating depression after basal ganglia ischemic stroke and to examine whether such effects are related to restoration of white matter integrity.

Methods and analysis: Sixty-six participants will be recruited from Zhujiang Hospital, Nanfang Hospital and Sichuan Bayi Rehabilitation Hospital and randomized with a 1:1 ratio to receive active rTMS treatment or sham rTMS treatment in addition to routine supportive treatments. The data will be collected at 0, 2 and 4 weeks after the commencement of the treatment. The primary outcome is the measurement of 24-item Hamilton Depression Rating Scale (HAMD-24) scores, and the secondary

For peer review only

outcomes include diffusion tensor imaging (DTI) results and the results of neuropsychological tests including National Institutes of Health Stroke Scale (NIHSS), Activities of Daily Living Scale (ADLs), Montreal Cognitive Assessment (MoCA), Clinical Global Impressions scales (CGI), Aphsia Battery in Chinese (ABC), Social Support Revalued Scale (SSRS) and Medical Coping Modes Questionnaire (MCMQ).

Ethics and dissemination: This study has been approved by the Ethics Committee of Zhujiang Hospital of Southern Medical University. The findings will be disseminated by publication in a peer-reviewed journal and by presentation in international conferences.

Trial registration: ClinicalTrials.gov, Identifier: NCT03159351.

Strengths and limitations of this study

- We focus on the efficacy of rTMS in patients with post-stroke depression (PSD) with the purpose of providing a mature approach to the treatment of PSD.
- We explore the effect of high-frequency stimulation on the left and low-frequency stimulation on the right dorsolateral prefrontal cortex in patients with PSD, aiming to afford a higher level of evidence for the stimulation parameter settings in rTMS treatment with PSD patients.
- A non-invasive DTI based on objective assessment of nerve fibers is used to evaluate with the therapeutic effect of rTMS on PSD.
- The patients will receive only short-term treatment and will be followed up for 1 month.
- Participants from only two provinces in China will be included.

INTRODUCTION

Background and rationale

Stroke is a global health problem and in 2015, the number of deaths related to stroke reached 6.326 million around the world, among which 45.89% were attributed to ischemic stroke.[1] Depression occurs frequently in the stroke survivors and has a significant impact on post-stroke rehabilitation.[2] The conventional treatment of post-stroke depression relies on supportive therapy and medications, but a proportion of patients can remain treatment-refractory.[3] Studies have shown that repetitive transcranial magnetic stimulation (rTMS) had a definite antidepressant effect on major depression,[4] and a recent meta-analysis also suggests the beneficial effects of rTMS on PSD, but such studies should be carefully examined for their heterogeneity and potential biases.[5] To our knowledge, currently no studies have been reported to examine the effect of rTMS on PSD in China, and the mechanism by which rTMS improves PSD remains to be clarified. In addition, the stimulation parameter settings in rTMS treatment still await standardization, and it is difficult to determine the optimal frequency (between 5 Hz to 20 Hz) in high-frequency rTMS of the left dorsolateral prefrontal cortex (DLPFC).

The basal ganglia area is the predilection site of stroke. This area, which is not an exact anatomical concept but one originating from neuroimaging, consists of the basal

For peer review only - http://bmjopen.bmj.com/site/about/guidelines.xhtml

ganglia, the capsula interna and the surrounding white matter;[6] in other words, the basal ganglia area covers most of the subcortical structures that constitute the limbic-cortical-striatal-pallidal-thalamic (LCSPT) circuit.[7] The limbic system is known to be closely related with emotional regulation,[8] and as the LCSPT circuit is the most important part of the limbic system, LCSPT dysfunctions are thought to contribute to the occurrence of depression.[9] Investigators reported a high incidence of depression after cerebral infarction in the basal ganglia area.[10] Our group previously found that structural damage of the LCSPT circuit, as a result of white matter damage following cerebral infarction in the basal ganglia area, was associated with a high likeliness of PSD.[11] The nuclei in the LCSPT circuit are connected via nerve fibers, and damage to these nerve fibers disrupts the integrity of the white matter as shown by magnetic resonance imaging (MRI).[12]

As numerous studies have demonstrated the beneficial effect of rTMS treatment on white matter integrity,[13-15] we hypothesized that the antidepressant effect of rTMS in patients with depression following cerebral infarction in the basal ganglia area is related to its action on the LCSPT circuit. The aim of this multicenter, randomized, controlled trial is to assess the effectiveness of rTMS in treating PSD and investigate the role of the LCSPT circuit in the therapeutic effect of rTMS for PSD.

METHODS

Study setting

The study will be conducted in two university hospitals, namely Zhujiang Hospital and Nanfang Hospital affiliated to Southern Medical University (Guangzhou, China) and in Sichuan Provincial Bayi Rehabilitation Hospital, a general hospital in Chengdu, China. The patients will be assessed for enrollment, and eligible patients will be hospitalized for treatment in the departments of neurology in the 3 hospitals.

Trial design

This randomized, double-blind, placebo-controlled multicenter superiority trial is designed to recruit two parallel groups, and randomization will be performed with block randomization in a 1:1 allocation ratio. The study design is shown in the flow chart in Figure 1.

Participant enrollment and eligibility criteria

The participants will be recruited by advertisement on the Internet and using posters in hospitals. The potentially eligible patients will be screened for enrollment according to the inclusion and exclusion criteria listed in **Table 1**.

Table 1 Inclusion and exclusion criteria

Criteria	Description
**Inclusion criteria**	1. First-time ischemic stroke with clinical and MRI or CT findings of basal ganglia ischemic stroke and a DSM-IV diagnosis of depression due to stroke (ICD-10-CM code 293.83[F06.32]);<br>2. Aged 25-75 years with a recent (from 3 weeks to 3 months) ischemic stroke;<br>3. Clear signs of neurological deficits in the acute phase;<br>4. Clear consciousness;

For peer review only

	5. Right-handedness.
**Exclusion criteria**	1. Aphasia or severe cognitive impairment, severe hearing impairment, or severe language comprehension deficits due to other causes;<br>2. Other cerebral diseases such as Parkinson's disease, encephalitis, dementia, multiple sclerosis, head injury, etc.;<br>3. Severe systemic disease or ongoing neoplasia;<br>4. Ongoing post-operative recovery;<br>5. Prior history of depressive disorders or major trauma within 1 year, severe depression or any other severe mental disorders;<br>6. Current or prior antidepressant use for any reason.<br>7. Addiction to drugs, alcohol or other substances;<br>8. Contraindications of MRI scan and rTMS treatment such as pacemaker implantation, a history of epilepsy, major head trauma, and seizures, etc;<br>9. Pregnant or breast-feeding women;<br>10. Participation in other clinical research projects;<br>11. Refusal to sign the informed consent of this study.

Ethical considerations

This study has been approved by the Ethics Committee of Zhujiang Hospital of Southern Medical University. The methods, purpose and potential risks of this study will be fully explained to the participants and their family members. Each patient will be asked to sign 3 informed consents before participating in this study.

Sample size calculation

The sample size was calculated based on the data of Valliengo et al,[16] who reported a response rate of 0.041 in patients receiving sham rTMS treatment and a rate of 0.375 in patients having active rTMS treatment. Considering the low efficiency of sham rTMS treatment, we calculated the sample size using the following formula:

$$n_1 = n_2 = \frac{\left(Z_{\alpha/2} + Z_{\beta}\right)^2}{2\left(\sin^{-1}\sqrt{p_2} - \sin^{-1}\sqrt{p_1}\right)^2}。$$

The result showed that a sample size of 26 in each group was sufficient by assuming a two-tailed significance level of 5% with a statistical power of 90%. We estimate that the dropout rate will be less than 25%, and considering the need of randomisation, we assume that at least 33 participants are required in each group.

Randomisation and blinding

For randomisation of the patients, random numbers will be generated using SAS9.4 software by statisticians based on a randomized block design with a block length of 6. The magnetic stimulation coils for active and sham treatments have identical appearances and will be numbered by a statistician. The random numbers will be printed and sent to each trial center in sealed envelopes. Upon enrollment of an eligible patient, the investigator opens the envelope to reveal the number of the magnetic stimulation coil to be used, without knowing whether the patient receives active or sham treatment. In this double-blind trial, both the participants and

For peer review only

investigators are blinded to the nature of treatment (active or sham).

Interventions

The participants enrolled in this trial will receive either active or sham rTMS treatment in addition to routine supportive treatments including antiplatelet agents, blood pressure control, neurotrophic therapy, physical therapy, and cognitive rehabilitation prescribed based on the condition of the individual participant. The enrolled patients will not receive any other antidepressants, anxiolytics or hypnotics treatments that may have an impact on depression treatment during the study. All the participants will receive psychiatric and neuropsychological tests and magnetic resonance imaging (MRI) examination at the time of enrollment and at the end of treatment.

rTMS will be performed using a Mag Pro R30 device (Mag Venture Company, Frum, Denmark) via 121-mm circular coils (MCF-125). The participants will receive active or sham treatment 5 days per week for 4 consecutive weeks. The motor threshold (MT) was defined as the lowest stimulation intensity capable of inducing at least 50 μV peak-to-peak amplitude of motor evoked potentials in at least half of 10 trials. The detailed parameters of the protocols of active rTMS treatment are listed in **Table 2**.

Table 2 Active rTMS treatment protocols

Localization	Frequency	Intensity	Times/ train	trains	Duration	Total times
Left DLPFC	10 Hz	110%MT	200	10	40 seconds	20
Right DLPFC	1 Hz	100%MT	30	10	10 seconds	20

Magnetic resonance imaging

Upon enrollment and at the end of this trial, the patient will receive MRI examination using a 3.0T PHILIPS Magnetic Trio Tim scanner. For the standard workup, MRI will be performed using the following scan parameters for different sequences: for T1-weighted sequence, time of echo (TE)=20 millisecond (ms), time of repetition (TR)=2000 ms, number of signal averaged (NSA)=1, slice=32, slice thickness=3 mm; for T2-weighted sequence, TE=80 ms, TR=3000 ms, NSA=2, slice=32, slice thickness=3 mm; for FLAIR sequence, TE=80 ms, TR=3000 ms, NSA=2, slice=32, slice thickness=3 mm; for diffusion tensor imaging (DTI) using single short echo planar imaging (SE-EPI), TE=68 ms, TR=2300 ms, NSA=1, slice=32, slice thickness=3 mm, b_0=0, b_1=1000, matrix=512*512. Once the original data have been obtained, an experienced imaging physician will select the caudate nucleus, lentiform nucleus, dorsal medial nucleus of thalamus, and nucleus of the thalamus as the region of interests (ROIs) at the imaging layer of the basal ganglia. The three-dimensional fiber bundle imaging technique will be used to display the fibrous pathway of the dorsal basal ganglia system and the fibrous pathway of the ventral basal ganglia system. The DTI parameters will be obtained, including the fractional anisotropy (FA), apparent diffusion coefficient (ADC), and neural fiber number (NFN). The MRI scanners at the 3 centres will be tested for homogeneity before the trial, while ROI

For peer review only

selection and DTI data acquisition will be performed by an experienced radiologist using PHILIP 2.1 software.

Outcome assessment

Baseline assessment

The demographic data will be collected from the patients, including age, gender, height, body weight, education level, occupation, and marital status. The vital signs will be measured including body temperature, pulse, respiration, and blood pressure. The lesion characteristics including lateralization and the number and size of the lesion will be recorded. The data including the past history and treatment prescription, levels of blood lipids and blood glucose, liver function index and renal function index will be collected before randomisation by reviewing the medical records of the patients.

Primary efficacy parameters

The primary efficacy parameter is the change in HAMD-24 scores from the baseline at the end of the treatment. We plan to measure the response rate and remission rate as the primary efficacy parameters, determined by the changes in HAMD-24 scores from baseline at the end of the treatment. The response to treatment is defined as an at least 50% reduction of the HAMD-24 total score, with a final HAMD-24 score below 9. Remission is defined as a reduction of the HAMD-24 total score by at least 50% from the baseline. As a valid and reliable scale for assessing the severity of depression, HAMD scale has been widely used in studies of PSD and shows a good sensitivity in assessing treatment responses. HAMD-24 scale consists of subscales that examine interest, anxiety, hopelessness, sleep status, and self-abasement, etc., and a higher score suggests a poorer condition of mental disorder.[17] HAMD-24 scale will be scored for 3 times at 0, 2, and 4 weeks after the commencement of the treatment.

Secondary outcome measures

DTI results

Nerve fibers are important components of the LCSPT circuit apart from the cerebral cortex, corpus striatum and thalami. The nuclei are connected by abundant nerve fibers. DTI is a special sequence of MRI, which is currently the best non-invasive method to observe and track the nerve tracts. We will assess the DTI parameters of the patients including the fractional anisotropy (FA), apparent diffusion coefficient (ADC), neural fiber number (NFN) collected upon enrollment and at the end of the trial.

Neuropsychological tests

The neuropsychological status of the patients will be examined using the following scales or questionnaires: National Institutes of Health Stroke Scale (NIHSS), Activities of Daily Living Scale (ADLs), Montreal Cognitive Assessment (MoCA), which are widely used to evaluate neurological functions; Clinical Global Impressions scales (CGI), which is commonly used to measure the global symptom severity and treatment response for patients with mental disorders; Aphasia Battery in Chinese (ABC), which is used to estimate the linguistic functions of Chinese patients; and Social Support Revalued Scale (SSRS) and Medical Coping Modes Questionnaire score (MCMQ), which evaluate how social support influences emotions of the

For peer review only

patients. All the tests will be performed at 0, 2 and 4 weeks after the commencement of the treatment.

Assessment of adverse events

All the adverse events (AEs) will be reported and recorded in the case report form (CRF) by investigators. The investigators will record the date of the occurrence, duration, and severity of the AEs, along with the treatments and consequences. Cases of serious AEs will be immediately reported to the project leader and the ethics committee, and the latter will evaluate whether the patient should receive further treatment. We will discontinue the allocated interventions once the disease has significant worsening. Those participants who suffer harm from this trial will be provided with compensation.

Quality control and data management

The trial protocol has been revised several times by experts in neurology, rehabilitation, medical imaging, statistics and psychology. All the investigators have participated in the training sessions to ensure that they fully understand the trial protocols and the standard procedures before the study. Inspectors will be nominated to regularly visit each research center to monitor the implementation of the protocols and assess the compliance of the participants and investigators. The data of CRFs will be double-entered by statisticians to ensure the accuracy of the data. Statisticians in our team will be responsible for data management and analysis. To improve the participants' adherence to this study, we will select the participants rigorously and cover the costs of neuropsychological assessment and MRI. Meanwhile, At the end of the patient's treatment, the patient will be informed about the follow-up, and a telephone call will be made in the fourth week after the end-point to remind them follow-up.

Statistical analysis

The quantitative data, including the demographic indicators, laboratory test result, MRI results and neuropsychological test results, will be expressed as numbers, means with standard deviation or median with range or quartile range as appropriate. These data will be compared using independent-sample *t*-test or Rank sum test depending on whether the data are normally distributed. The qualitative data, including the response and emission rates and incidences of the AEs, will be expressed as frequency or frequency rate and compared using a χ^2 test between the two groups. The missing data will be described in terms of exit data, exit reason, treatment arm, frequencies and percentage of missing data in each group and the data imbalances will be evaluated by $\chi 2$ test of Fisher's exact test. All the data will be double-entered into Epidata software and analysed using SAS software by statisticians blinded to the participants' allocation. A *P* value less than 0.05 will be considered to indicate a statistically significant difference.

Monitoring

All personal information collected from the potential and enrolled participants will be stored in the medical record management system maintained by the Information Security Department of each hospital to ensure the confidentiality. Investigators will record the research information on the CRFs and identify the CRFs with only initials

For peer review only

and participation number instead of the participants' real identity. The research information has been detailed above, including neuropsychological test, MRI results, baseline parameters and so on. All the CRFs will be kept securely in a locked area. The data managers, study statisticians, and investigators will have access to the final trial dataset. The data managers and study statisticians only access the data for data management and statistical analysis, will not be involved in the clinical treatment and psychological assessment. An annual audit by the Delegation of Research Department of Southern Medical University is provided.

DISCUSSION

The conventional treatments of PSD include selective serotonin reuptake inhibitors (SSRIs),[18] tricyclic antidepressants (TCA)[19] and electroconvulsive therapy (ECT)[20], which are not always effective. Recent evidence has shown that rTMS has antidepressant actions [21,22] and produces therapeutic effects comparable to those of ECT.[23] In spite of the various hypotheses that have been proposed, the exact mechanism underlying the antidepressant effect of rTMS remains unknown. [24-26]

In this trial, we determined the frequency and intensity of rTMS treatment based on previous studies reporting the positive antidepressant effect of the treatment,[27-29] and chose the settings of pulses and sessions according to an evidence-based guideline.[4] As both high-frequency rTMS of the left DLPFC and low-frequency rTMS of the right DLPFC are recommended in major depression treatment, and considering that patients with PSD have more brain structural changes than those with primary depression, we chose bilateral DLPFC stimulation for rTMS treatment in this study.

White matter changes play important roles in the development of many neuropsychiatric diseases,[30] including cognitive and emotional disorders. [31] As a non-invasive imaging technique, DTI provides an important modality for evaluating white matter integrity in vivo. In DTI, the parameter FA can sensitively reflect the integrity of the white matter fibers while the ADC value allows sensitive detection of changes in the density and number of the nerve fibers.[32] Numerous studies have reported reduced FA values in the brain of patients with depression,[33,34] and the structural and functional brain abnormalities, particularly in the LCSPT system, are thought to be associated with the occurrence of depression.[35] Our previous study has confirmed that the occurrence of PSD was associated with neurological functional deficits following basal ganglia infarction, and the depression level was correlated with the reductions in the FA values of the bilateral pallidum and the left putamen.

DTI offers an important means for evaluating the therapeutic effect in patients with PSD. Allendorfer et al compared the DTI data collected before and after a 10-day iTBS treatment from 8 patients with post-stroke aphasia and found significant increments of the FA values near the inferior and superior frontal gyri and the anterior corpus callosum after the treatment.[15] Liao et al reported that in patients with treatment-resistant depression (TRD), the FA value decreased significantly in the white matter in the left middle frontal gyrus, and 4 weeks of active rTMS treatment resulted in obvious improvement of the decreased FA values.[36] Lyden et al also

For peer review only

confirmed the positive effect of a one-week-long electroconvulsive therapy (ECT) on major depression by reporting significant increases in the FA values in the bilateral anterior cingulum and forceps minor after the treatment.[37] Compared with major depression, PSD is associated with more severe structural and functional abnormalities of the brain, but currently researches evaluating white matter integrity in PSD patients using DTI remain insufficient. In this study, we will compare the DTI data before and after 4 weeks of rTMS treatment as a secondary outcome parameter to assess the effect of rTMS on white matter integrity in PSD patients.

This trial is expected to produce additional evidence to verify the effects of rTMS treatment on depression after ischemic stroke in the basal ganglia area; we also aim to explore the role of the LCSPT circuit in PSD. Findings from this study will add randomized controlled trial data of rTMS for PSD in Chinese patients, and contribute to the clinical efficacy evaluation of rTMS and also to the improvement of the neural loop theory.

Contributions SZ, SP, MS and LZ participated in the conception and design of the trial. AC, JZ, and LZ participated in planning the analysis of the data. YT, AC, and LY will be responsible for data collection and participant recruitment. AC, LY, SP, MS and LZ will participate in the treatment of participants. YT and LZ participated in drafting this manuscript. All the authors discussed, revised and approved the final manuscript.

Acknowledgements Authors appreciate the effort of all the people participating in this trail.

Sponsor contact information Trial sponsor: Southern Medical University. Contact information: Mr. Yan Zhang, E-mail:zjyykyc@126.com.

Sponsor and funder This funding source had no role in the design of this study and will not have any role during its execution, analyses, interpretation of the data, or decision to submit results.

Funding This study was supported by grants from the Training Program of the Clinical Research Plan of Southern Medical University (LC2016PY031).

Competing interests None declared.

Patient consent Obtained Obtained.

Ethics approval The ethics committee of Zhujiang Hospital of Southern Medical University approved the study (Identifier: 2016-SJNK-005).

Provenance and peer review Not commissioned; peer reviewed for ethical and funding approval prior to submission.

Dissemination policy The results of this original research will be disseminated by publication in a peer-reviewed journal and presentation at international conferences.

Trial status The trial is currently ongoing in the stage of participant enrollment; the recruitment of patients started on 1 June, 2017 and will be completed on 31 May, 2020.

Open Access This is an Open Access article distributed in accordance with the terms of the Creative Commons Attribution (CC BY 4.0) license, which permits others to distribute, remix, adapt and build upon this work, for commercial use, provided the

For peer review only

original works is properly cited. See: http://creativecommons.org/licenses/by/4.0/

REFERENCES

1 Global, regional, and national life expectancy, all-cause mortality, and cause-specific mortality for 249 causes of death, 1980-2015: a systematic analysis for the Global Burden of Disease Study 2015. *Lancet* 2016;388(10053):1459-1544.

2 Matsuzaki S, Hashimoto M, Yuki S, et al. The relationship between post-stroke depression and physical recovery. *J Affect Disord* 2015;176:56-60.

3 Mrazek DA, Hornberger JC, Altar CA, et al. A review of the clinical, economic, and societal burden of treatment-resistant depression: 1996-2013. *Psychiatr Serv* 2014;65(8):977-87.

4 Lefaucheur JP, Andre-Obadia N, Antal A, et al. Evidence-based guidelines on the therapeutic use of repetitive transcranial magnetic stimulation (rTMS). *Clin Neurophysiol* 2014;125(11):2150-206.

5 Shen X, Liu M, Cheng Y, et al. Repetitive transcranial magnetic stimulation for the treatment of post-stroke depression: A systematic review and meta-analysis of randomized controlled clinical trials. *J Affect Disord* 2017;211:65-74.

6 Naritomi H, Sawada T, Kuriyama Y, et al. Effect of chronic middle cerebral artery stenosis on the local cerebral hemodynamics. *Stroke* 1985;16(2):214-9.

7 Haber SN. The place of dopamine in the cortico-basal ganglia circuit. *Neuroscience* 2014;282:248-57.

8 Joyce JN. The dopamine hypothesis of schizophrenia: limbic interactions with serotonin and norepinephrine. *Psychopharmacology (Berl)* 1993;112(1 Suppl):S16-34.

9 Berton O, Nestler EJ. New approaches to antidepressant drug discovery: beyond monoamines. *Nat Rev Neurosci* 2006;7(2):137-51.

10 Wu RH, Li Q, Tan Y, et al. Depression in silent lacunar infarction: a cross-sectional study of its association with location of silent lacunar infarction and vascular risk factors. *Neurol Sci* 2014;35(10):1553-9.

11 Jia-shan TU, Zhen-hua L, Fan-heng H, et al. Evaluation of depression status following basal ganglia infarction by diffusion tensor magnetic resonance imaging. *Chinese Journal of Neurology* 2012;45(8):590-594.

12 Nugent AC, Davis RM, Zarate CJ, et al. Reduced thalamic volumes in major depressive disorder. *Psychiatry Res* 2013;213(3):179-85.

13 Kozel FA, Johnson KA, Nahas Z, et al. Fractional anisotropy changes after several weeks of daily left high-frequency repetitive transcranial magnetic stimulation of the prefrontal cortex to treat major depression. *J ECT* 2011;27(1):5-10.

14 Yang W, Liu TT, Song XB, et al. Comparison of different stimulation parameters of repetitive transcranial magnetic stimulation for unilateral spatial neglect in stroke patients. *J Neurol Sci* 2015;359(1-2):219-25.

15 Allendorfer JB, Storrs JM, Szaflarski JP. Changes in white matter integrity follow excitatory rTMS treatment of post-stroke aphasia. *Restor Neurol Neurosci* 2012;30(2):103-13.

16 Valiengo LC, Goulart AC, de Oliveira JF, et al. Transcranial direct current stimulation for the treatment of post-stroke depression: results from a randomised, sham-controlled, double-blinded trial. *J Neurol Neurosurg Psychiatry* 2016.

17 Williams JB, Kobak KA, Bech P, et al. The GRID-HAMD: standardization of the Hamilton Depression Rating Scale. *Int Clin Psychopharmacol* 2008;23(3):120-9.

For peer review only

18 Mead GE, Hsieh CF, Hackett M. Selective serotonin reuptake inhibitors for stroke recovery. *JAMA* 2013;310(10):1066-7.
19 Capaldi VN, Wynn GH. Post stroke depression: treatments and complications in a young adult. *Psychiatr Q* 2010;81(1):73-9.
20 Berggren A, Gustafson L, Hoglund P, et al. A long-term follow-up of clinical response and regional cerebral blood flow changes in depressed patients treated with ECT. *J Affect Disord* 2014;167:235-43.
21 Kim BR, Kim DY, Chun MH, et al. Effect of repetitive transcranial magnetic stimulation on cognition and mood in stroke patients: a double-blind, sham-controlled trial. *Am J Phys Med Rehabil* 2010;89(5):362-8.
22 Levkovitz Y, Isserles M, Padberg F, et al. Efficacy and safety of deep transcranial magnetic stimulation for major depression: a prospective multicenter randomized controlled trial. *World Psychiatry* 2015;14(1):64-73.
23 Ren J, Li H, Palaniyappan L, et al. Repetitive transcranial magnetic stimulation versus electroconvulsive therapy for major depression: a systematic review and meta-analysis. *Prog Neuropsychopharmacol Biol Psychiatry* 2014;51:181-9.
24 Yulug B, Hanoglu L, Tavli AM, et al. The Brain Protective Effect of rTMS (Repetitive Transcranial Magnetic Stimulation) in Depression: A Mini-Review in Animal Studies. *Med Chem* 2016;12(6):500-5.
25 Wang HN, Wang L, Zhang RG, et al. Anti-depressive mechanism of repetitive transcranial magnetic stimulation in rat: the role of the endocannabinoid system. *J Psychiatr Res* 2014;51:79-87.
26 Peng H, Zheng H, Li L, et al. High-frequency rTMS treatment increases white matter FA in the left middle frontal gyrus in young patients with treatment-resistant depression. *J Affect Disord* 2012;136(3):249-57.
27 Jorge RE, Robinson RG, Tateno A, et al. Repetitive transcranial magnetic stimulation as treatment of poststroke depression: a preliminary study. *Biol Psychiatry* 2004;55(4):398-405.
28 Herbsman T, Avery D, Ramsey D, et al. More lateral and anterior prefrontal coil location is associated with better repetitive transcranial magnetic stimulation antidepressant response. *Biol Psychiatry* 2009;66(5):509-15.
29 Avery DH, Holtzheimer PR, Fawaz W, et al. A controlled study of repetitive transcranial magnetic stimulation in medication-resistant major depression. *Biol Psychiatry* 2006;59(2):187-94.
30 Filley CM. The behavioral neurology of cerebral white matter. *Neurology* 1998;50(6):1535-40.
31 Filley CM, Fields RD. White matter and cognition: making the connection. *J Neurophysiol* 2016;116(5):2093-2104.
32 Peng H, Zheng H, Li L, et al. High-frequency rTMS treatment increases white matter FA in the left middle frontal gyrus in young patients with treatment-resistant depression. *J Affect Disord* 2012;136(3):249-57.
33 Kozel FA, Johnson KA, Nahas Z, et al. Fractional anisotropy changes after several weeks of daily left high-frequency repetitive transcranial magnetic stimulation of the prefrontal cortex to treat major depression. *J ECT* 2011;27(1):5-10.
34 Peng H, Zheng H, Li L, et al. High-frequency rTMS treatment increases white matter FA in the left middle frontal gyrus in young patients with treatment-resistant depression. *J Affect Disord* 2012;136(3):249-57.
35 Sheline YI. Neuroimaging studies of mood disorder effects on the brain. *Biol Psychiatry* 2003;54(3):338-52.
36 Liao Y, Tang J, Ma M, et al. Frontal white matter abnormalities following chronic ketamine use: a

For peer review only

diffusion tensor imaging study. *Brain* 2010;133(Pt 7):2115-22.
37 Lyden H, Espinoza RT, Pirnia T, et al. Electroconvulsive therapy mediates neuroplasticity of white matter microstructure in major depression. *Transl Psychiatry* 2014;4:e380.

Figure legends

Figure1：flow chart of the trial design.

For peer review only

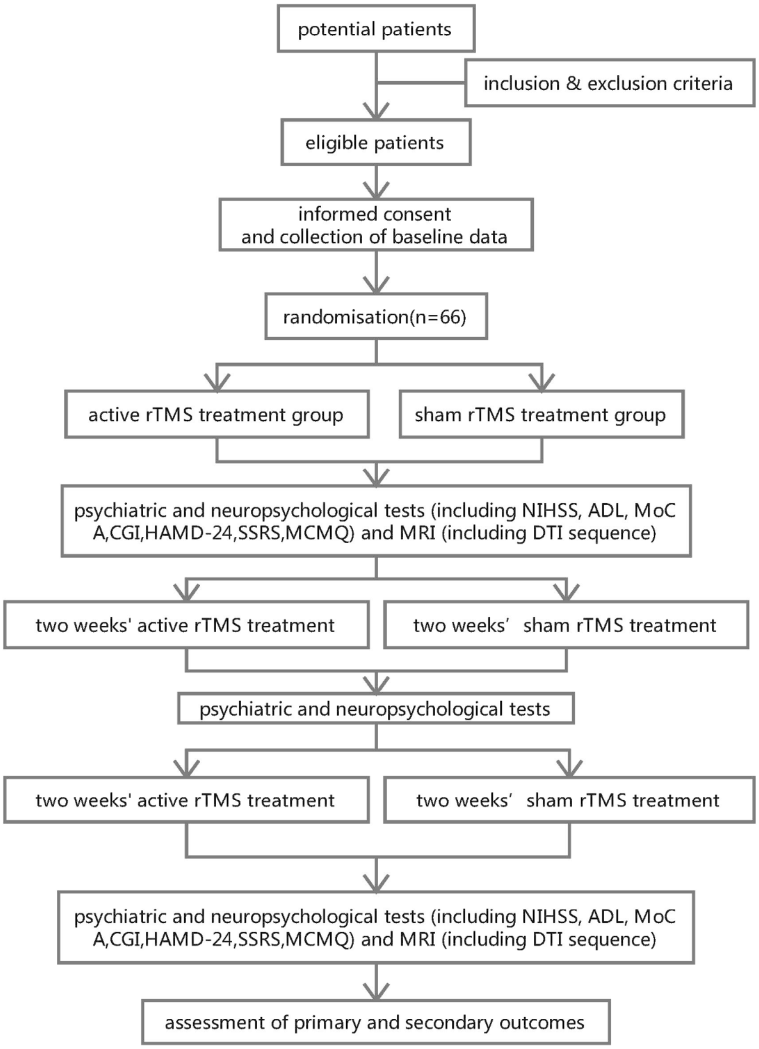

Figure1: Flow chart of the study design

173x228mm (300 x 300 DPI)

SPIRIT

STANDARD PROTOCOL ITEMS: RECOMMENDATIONS FOR INTERVENTIONAL TRIALS

SPIRIT 2013 Checklist: Recommended items to address in a clinical trial protocol and related documents*

Section/item	ItemNo	Description	Description
**Administrative information**			
Title	1	Descriptive title identifying the study design, population, interventions, and, if applicable, trial acronym	Repetitive transcranial magnetic stimulation for depression after basal ganglia ischemic stroke: protocol for a multicenter, randomized, double-blind, placebo-controlled trial. Page1
Trial registration	2a	Trial identifier and registry name. If not yet registered, name of intended registry	ClinicalTrials.gov, Identifier: NCT03159351. Page2
	2b	All items from the World Health Organization Trial Registration Data Set	
Protocol version	3	Date and version identifier	19/02/2017
Funding	4	Sources and types of financial, material, and other support	Training Program of the Clinical Research Plan of Southern Medical University (LC2016PY031). Page2
Roles and responsibilities	5a	Names, affiliations, and roles of protocol contributors	SZ, SP, MS and LZ participated in the conception and design of the trial. AC, JZ ,and LZ participated in planning the analysis of the data. YT, AC, LY ,participated in data collection, participants recruitment. AC, LY, SP, MS and LZ participated in the treatment of participants. YT and LZ participated in drafting the manuscript. All the authors discussed, revised and approved the final manuscript. Page9
	5b	Name and contact information for the trial sponsor	Yan Zhang, the administrator of the Clinical Research Plan of Southern Medical University，E-mail: zjyykyc@126.com. Page9

For peer review only - http://bmjopen.bmj.com/site/about/guidelines.xhtml

5c	Role of study sponsor and funders, if any, in study design; collection, management, analysis, and interpretation of data; writing of the report; and the decision to submit the report for publication, including whether they will have ultimate authority over any of these activities	This funding source had no role in the design of this study and will not have any role during its execution, analyses, interpretation of the data, or decision to submit results. Page9
5d	Composition, roles, and responsibilities of the coordinating centre, steering committee, endpoint adjudication committee, data management team, and other individuals or groups overseeing the trial, if applicable (see Item 21a for data monitoring committee)	NA

Introduction

For peer review only - http://bmjopen.bmj.com/site/about/guidelines.xhtml

Background and rationale	6a	Description of research question and justification for undertaking the trial, including summary of relevant studies (published and unpublished) examining benefits and harms for each intervention	Stroke is a global health problem and in 2015, the number of deaths related with stroke reached 6.326 million around the world, among which 45.89% were attributed to ischemic stroke Depression occurs frequently in the stroke survivors and has a significant impact on post-stroke rehabilitation. The conventional treatment of post-stroke depression (PSD) relies on supportive therapy and medications, but a proportion of patients can remain treatment-refractory. Studies has shown that repetitive transcranial magnetic stimulation (rTMS) has a definite antidepressant effect on major depression, and a recent meta-analysis also suggests the beneficial effects of rTMS on PSD, but such studies should be carefully examined for their heterogeneity and potential biases. To our knowledge, currently no studies have been reported to examine the effect of rTMS on PSD in China, and the mechanism by which rTMS improves PSD remains to be clarified. In addition, the stimulation parameter settings in rTMS treatment still awaits standardization, and it is difficult to determine the optimal frequency (between 5 Hz to 20 Hz) in high-frequency rTMS of the left dorsolateral prefrontal cortex (DLPFC). The basal ganglia area is the predilection site of stroke. The basal ganglia area, which is not an exact anatomical concept but one originating from neuroimaging, consists of the basal ganglia, the capsula interna and the surrounding white matter ; in other words, the basal ganglia area covers most of the subcortical structures that constitute the limbic-cortical-striatal-pallidal-thalamic (LCSPT) circuit. The limbic system is known to be closely related with emotional regulation, and as the LCSPT circuit is the most important part of the limbic system, LCSPT dysfunctions are thought to contribute to the occurrence of depression. Investigators reported a high incidence of depression after cerebral infraction in the basal ganglia area. Our group previously found that structural damage of the LCSPT circuit, as a result of white matter damage following cerebral infraction in the basal ganglia area, was associated with a high likeliness of PSD. The nuclei in the LCSPT circuit are connected via nerve fibers, and damage of these nerve fibers disrupted the integrity of the white matter as shown by magnetic resonance imaging (MRI). Page2-3

For peer review only

	6b	Explanation for choice of comparators	To our knowledge, currently no studies have been reported to examine the effect of rTMS on PSD in China, and the mechanism by which rTMS improves PSD remains to be clarified. In addition, the stimulation parameter settings in rTMS treatment still awaits standardization, and it is difficult to determine the optimal frequency (between 5 Hz to 20 Hz) in high-frequency rTMS of the left dorsolateral prefrontal cortex (DLPFC). Page2<br>We determined the optimal frequency and intensity in rTMS treatment based on previous studies reporting positive antidepressant effect of the treatment, and chose the settings of pulses and sessions according to an evidence-based guideline. As both high-frequency rTMS of the left DLPFC and low-frequency rTMS of the right DLPFC are recommended in major depression treatment and considering that patients with PSD have more brain structural changes than those with primary depression, we chose bilateral rTMS of the DLPFC for treatment in this study. Page 8
Objectives	7	Specific objectives or hypotheses	We hypothesized that the antidepressant effect of rTMS in patients with depression following cerebral infarction in the basal ganglia area is related to its action on the LCSPT circuit. The aim of this multicenter, randomized, controlled trial is to assess the effectiveness of rTMS in treating PSD and investigate the role of the LCSPT circuit in the therapeutic effect of rTMS for PSD. Page3
Trial design	8	Description of trial design including type of trial (eg, parallel group, crossover, factorial, single group), allocation ratio, and framework (eg, superiority, equivalence, noninferiority, exploratory)	This randomized, double-blind, placebo-controlled multicenter superiority trial is designed to recruit two parallel groups, and randomization will be performed with block randomization in a 1:1 allocation ratio. Page3
**Methods: Participants, interventions, and outcomes**			
Study setting	9	Description of study settings (eg, community clinic, academic hospital) and list of countries where data will be collected. Reference to where list of study sites can be obtained	The study will be conducted in two university hospitals, namely Zhujiang Hospital and Nanfang Hospital affiliated to Southern Medical University (Guangzhou, China) and in Sichuan Provincial Bayi Rehabilitation Hospital, a general hospital in Chengdu, China. Page3

For peer review only - http://bmjopen.bmj.com/site/about/guidelines.xhtml

Eligibility criteria	10	Inclusion and exclusion criteria for participants. If applicable, eligibility criteria for study centres and individuals who will perform the interventions (eg, surgeons, psychotherapists)	Inclusion criteria:1. First-time ischemic stroke with clinical and MRI or CT findings of basal ganglia ischemic stroke and a DSM-IV diagnosis of depression due to stroke (ICD-10-CM code 293.83[F06.32]); 2. Aged 25-75 years with a recent (from 3 weeks to 3 months) ischemic stroke; 3. Clear signs of neurological deficits in the acute phase;4. Clear consciousness;5. Right-handedness.<br>Exclusion criteria:1.Aphasia or severe cognitive impairment, severe hearing impairment, or severe language comprehension deficits due to other causes; 2.Other cerebral diseases such as Parkinson's disease, encephalitis, dementia, multiple sclerosis, head injury, etc.; 3.Severe systemic disease or ongoing neoplasia; 4.Ongoing post-operative recovery; 5.Prior history of depressive disorders or major trauma within 1 year, severe depression or any other severe mental disorders; 6.Current or prior antidepressant use for any reason. 7.Addiction to drugs, alcohol or other substances; 8.Contraindications of MRI scan and rTMS treatment such as pacemaker implantation, a history of epilepsy, major head trauma, and seizures, etc; 9.Pregnant or breast-feeding women; 10.Participation in other clinical research projects; 11.Refusal to the sign informed consent of this study. Page3-4
Interventions	11a	Interventions for each group with sufficient dcetail to allow replication, including how and when they will be administered	rTMS will be performed using a Mag Pro R30 device (Mag Venture Company, Frum, Denmark) via 121mm circular coils (MCF-125). The participants will receive active or sham treatment 5 days per week for 4 consecutive weeks. The motor threshold (MT) was defined as the lowest stimulation intensity capable of inducing at least 50 μV peak-to-peak amplitude of motor evoked potentials in at least half of 10 trials. The detailed parameters of the protocols of active rTMS treatment are listed in Table 2. Page5
	11b	Criteria for discontinuing or modifying allocated interventions for a given trial participant (eg, drug dose change in response to harms, participant request, or improving/worsening disease)	We will discontinue the allocated interventions once the disease has significant worsening. Page7

For peer review only - http://bmjopen.bmj.com/site/about/guidelines.xhtml

	11c	Strategies to improve adherence to intervention protocols, and any procedures for monitoring adherence (eg, drug tablet return, laboratory tests)	To improve adherence to intervention protocols, we will select the participants rigorously and compensate the cost of neuropsychological assessment and MRI. Page7
	11d	Relevant concomitant care and interventions that are permitted or prohibited during the trial	The participants enrolled in this trial will receive either active or sham rTMS treatment in addition to routine supportive treatments including antiplatelet agents, blood pressure control, neurotrophic therapy, physical therapy, and cognitive rehabilitation prescribed based on the condition of the individual participant and will not receive any other antidepressants, anxiolytics or hypnotics treatments that may have an impact on depression treatment during the study. All the participants will receive psychiatric and neuropsychological tests and MRI examination at the time of enrolment and at the end of treatment. Page5
Outcomes	12	Primary, secondary, and other outcomes, including the specific measurement variable (eg, systolic blood pressure), analysis metric (eg, change from baseline, final value, time to event), method of aggregation (eg, median, proportion), and time point for each outcome. Explanation of the clinical relevance of chosen efficacy and harm outcomes is strongly recommended	The primary outcome is the measurement of 24-item Hamilton Depression Rating Scale (HAMD-24) scores, and the secondary outcomes include diffusion tensor imaging (DTI) results and the results of neuropsychological tests including National Institutes of Health Stroke Scale (NIHSS), Activities of Daily Living Scale (ADLs), Montreal Cognitive Assessment (MoCA), Clinical Global Impressions scales(CGI), Aphsia Battery in Chinese (ABC), Social Support Revalued Scale (SSRS) and Medical Coping Modes Questionnaire (MCMQ). Page6
Participant timeline	13	Time schedule of enrolment, interventions (including any run-ins and washouts), assessments, and visits for participants. A schematic diagram is highly recommended (see Figure)	See Figure 1.

For peer review only - http://bmjopen.bmj.com/site/about/guidelines.xhtml

Sample size	14	Estimated number of participants needed to achieve study objectives and how it was determined, including clinical and statistical assumptions supporting any sample size calculations	The primary efficacy parameter is the change in HAMD-24 scores from the baseline to the end of the treatment. The sample size was calculated based on the data of Valliengo et al[19], who reported a response rate of 0.041 in patients receiving sham rTMS treatment and a rate of 0.375 in patients having active rTMS treatment. Considering the low efficiency of sham rTMS treatment, we calculated the sample size using the following formula: $n_1 = n_2 = \frac{(Z_{\alpha/2} + Z_\beta)^2}{2(\sin^{-1}\sqrt{p_2} - \sin^{-1}\sqrt{p_1})^2}$ 。 The result showed that a sample size of 26 in each group was sufficient by assuming a two-tailed significance level of 5% and a statistical power of 90%. We estimate that the dropout rate will be less than 25%, and considering the need of randomisation, we assume that at least 33 participants are required in each group. Page4
Recruitment	15	Strategies for achieving adequate participant enrolment to reach target sample size	The participants will be recruited by advertisement on Internet and using posters in hospitals. Page3

Methods: Assignment of interventions (for controlled trials)

Allocation:

Sequence generation	16a	Method of generating the allocation sequence (eg, computer-generated random numbers), and list of any factors for stratification. To reduce predictability of a random sequence, details of any planned restriction (eg, blocking) should be provided in a separate document that is unavailable to those who enrol participants or assign interventions	Random numbers will be generated using SAS9.4 software by statisticians based on a randomized block design with a block length of 6. Page4

Allocation concealment mechanism	16b	Mechanism of implementing the allocation sequence (eg, central telephone; sequentially numbered, opaque, sealed envelopes), describing any steps to conceal the sequence until interventions are assigned	The magnetic stimulation coils for active and sham treatments have identical appearances and will be numbered by a statistician. The random numbers will be printed and sent to each trial center in sealed envelopes. Upon enrolment of an eligible patient, the investigator opens the envelope to receive the number of the magnetic stimulation coil to be used, without knowing whether the patient receives active or sham treatment. Page4
Implementation	16c	Who will generate the allocation sequence, who will enrol participants, and who will assign participants to interventions	The statisticians will generate the allocation sequence, investigators will enrol participants, and the project leader will assign participants intervention based on its allocation sequence. Page4
Blinding (masking)	17a	Who will be blinded after assignment to interventions (eg, trial participants, care providers, outcome assessors, data analysts), and how	In this double-blind trial, both the participants and investigators are blinded to the nature of treatment (active or sham). Page4
	17b	If blinded, circumstances under which unblinding is permissible, and procedure for revealing a participant's allocated intervention during the trial	We will discontinue the allocated interventions once the disease has significant worsening. Page7

Methods: Data collection, management, and analysis

w only

For peer review only - http://bmjopen.bmj.com/site/about/guidelines.xhtml

Data collection methods	18a	Plans for assessment and collection of outcome, baseline, and other trial data, including any related processes to promote data quality (eg, duplicate measurements, training of assessors) and a description of study instruments (eg, questionnaires, laboratory tests) along with their reliability and validity, if known. Reference to where data collection forms can be found, if not in the protocol	Baseline assessment<br>The demographic data will be collected from the patients, including age, gender, height, body weight, education level, occupation, and marital status. The vital signs will be measured including body temperature, pulse, respiration, and blood pressure. The lesion characteristics including the lateralization, number and size of the lesion.The data including the past history and treatment prescription, levels of blood lipids and blood glucose, liver function index and renal function index will be collected before randomisation by reviewing the medical records of the patients.<br>We plan to measure the response rate and remission rate as primary efficacy parameters, namely the changes in HAMD-24 scores from baseline to the end of the treatment. HAMD-24 scale will be scored for 3 times at 0, 2, and 4 weeks after the commencement of treatment.<br>DTI parameters and Neuropsychological tests are Secondary outcome measures. DTI parameters including the fractional anisotropy (FA), apparent diffusion coefficient (ADC), neural fiber number (NFN) will be scored at 0, and 4 weeks after the commencement of treatment.<br>neuropsychological tests including National Institutes of Health Stroke Scale (NIHSS), Activities of Daily Living Scale (ADLs), Montreal Cognitive Assessment (MoCA), Clinical Global Impressions scales(CGI), Aphsia Battery in Chinese (ABC), Social Support Revalued Scale (SSRS) and Medical Coping Questionnaire (MCMQ) will be implemented at 0, 2 and 4 weeks after the commencement of the treatment. Page6
	18b	Plans to promote participant retention and complete follow-up, including list of any outcome data to be collected for participants who discontinue or deviate from intervention protocols	To improve the participants’ adherence to this study, we will select the participants rigorously and cover the costs of neuropsychological assessment and MRI. Meanwhile, At the end of the patient's treatment, the patient will be informed about the follow-up, and a telephone call will be made in the fourth week after the end-point to remind them follow-up. Page7

For peer review only - http://bmjopen.bmj.com/site/about/guidelines.xhtml

Data management	19	Plans for data entry, coding, security, and storage, including any related processes to promote data quality (eg, double data entry; range checks for data values). Reference to where details of data management procedures can be found, if not in the protocol	The trial protocol has been revised for several times by experts in neurology, rehabilitation, medical imaging, statistics and psychology. All the investigators have participated in the training sessions to ensure that they fully understand the trial protocols and the standard procedures before the study. Inspectors will be nominated to regularly visit each research center to monitor the implementation of the protocols and assess the compliance of the participants and investigators. All the data will be recorded on CRF by the investigators. A staff member will be appointed to manage all the CRFs and ensure the completeness of the data. The data will be double-entered by statisticians to ensure the accuracy of the data. Statisticians in our team will be responsible for data management and analysis. Page7
Statistical methods	20a	Statistical methods for analysing primary and secondary outcomes. Reference to where other details of the statistical analysis plan can be found, if not in the protocol	The quantitative data, including the demographic indicators, laboratory test result, MRI results and neuropsychological test results, will be expressed as numbers, means with standard deviation or median with range or quartile range as appropriate. These data will be compared using independent-sample t-test or Rank sum test depending on whether the data are normally distributed. The qualitative data, including the response and emission rates and incidences of the AEs, will be expressed as frequency or frequency rate and compared using a χ2 test between the two groups. All the data will be double-entered into Epidata software and analysed using SAS software by statisticians blinded to the participants' allocation. A P value less than 0.05 will be considered to indicate a statistically significant difference. Page7
	20b	Methods for any additional analyses (eg, subgroup and adjusted analyses)	NA
	20c	Definition of analysis population relating to protocol non-adherence (eg, as randomised analysis), and any statistical methods to handle missing data (eg, multiple imputation)	Missing data will be described in terms of exit data, exit reason, treatment arm, frequencies and percentage of missing data in each group. The data imbalances will be evaluated by χ^2 test of Fisher's exact test. Page7

Methods: Monitoring

For peer review only - http://bmjopen.bmj.com/site/about/guidelines.xhtml

Data monitoring	21a	Composition of data monitoring committee (DMC); summary of its role and reporting structure; statement of whether it is independent from the sponsor and competing interests; and reference to where further details about its charter can be found, if not in the protocol. Alternatively, an explanation of why a DMC is not needed	The data managers and study statisticians only access the data for data management and statistical analysis, will not be involved in the clinical treatment and psychological assessment. Page7
	21b	Description of any interim analyses and stopping guidelines, including who will have access to these interim results and make the final decision to terminate the trial	No interim analyses is envisaged.
Harms	22	Plans for collecting, assessing, reporting, and managing solicited and spontaneously reported adverse events and other unintended effects of trial interventions or trial conduct	All adverse effects will be reported in the CRF. Page6
Auditing	23	Frequency and procedures for auditing trial conduct, if any, and whether the process will be independent from investigators and the sponsor	An annual audit by the Delegation of Research Department of Southern Medical University is provided. Page7
**Ethics and dissemination**			
Research ethics approval	24	Plans for seeking research ethics committee/institutional review board (REC/IRB) approval	The ethics committees of Zhujiang Hospital of Southern Medical University approved the study (Identifier: 2016-SJNK-005). Page9

For peer review only - http://bmjopen.bmj.com/site/about/guidelines.xhtml

Protocol amendments	25	Plans for communicating important protocol modifications (eg, changes to eligibility criteria, outcomes, analyses) to relevant parties (eg, investigators, REC/IRBs, trial participants, trial registries, journals, regulators)	NA
Consent or assent	26a	Who will obtain informed consent or assent from potential trial participants or authorised surrogates, and how (see Item 32)	The methods, purpose and potential risks of this study will be fully explained to all the participants and their family members. Each patient will be asked to sign 3 informed consents before participating in this study. Page4
	26b	Additional consent provisions for collection and use of participant data and biological specimens in ancillary studies, if applicable	NA
Confidentiality	27	How personal information about potential and enrolled participants will be collected, shared, and maintained in order to protect confidentiality before, during, and after the trial	All personal information collected from the potential and enrolled participants will be stored in the medical record management system maintained by the Information Security Department of each hospital to ensure the confidentiality. Investigators will record the research information on the CRFs and identify the CRFs with only initials and participation number instead of the participants' real identity. The research information has been detailed above, including neuropsychological test, MRI results, baseline parameters and so on. All the CRFs will be kept securely in a locked area. Page7
Declaration of interests	28	Financial and other competing interests for principal investigators for the overall trial and each study site	Authors have no conflicts of interest to declare. Page9
Access to data	29	Statement of who will have access to the final trial dataset, and disclosure of contractual agreements that limit such access for investigators	The data managers, study statisticians, and investigators will have access to the final trial dataset. The data managers and study statisticians only access the data for data management and statistical analysis, will not be involved in the clinical treatment and psychological assessment. Page7
Ancillary and post-trial care	30	Provisions, if any, for ancillary and post-trial care, and for compensation to those who suffer harm from trial participation	Those participants who suffer harm from this trial will be provided with compensation. Page7

For peer review only - http://bmjopen.bmj.com/site/about/guidelines.xhtml

For peer review only

Dissemination policy	31a	Plans for investigators and sponsor to communicate trial results to participants, healthcare professionals, the public, and other relevant groups (eg, via publication, reporting in results databases, or other data sharing arrangements), including any publication restrictions	The results of this original research will be disseminated by publication in a peer-reviewed journal and presentation at international conferences. Page9
	31b	Authorship eligibility guidelines and any intended use of professional writers	NA
	31c	Plans, if any, for granting public access to the full protocol, participant-level dataset, and statistical code	NA
**Appendices**			
Informed consent materials	32	Model consent form and other related documentation given to participants and authorised surrogates	Consent form has been validated by the ethical committee.
Biological specimens	33	Plans for collection, laboratory evaluation, and storage of biological specimens for genetic or molecular analysis in the current trial and for future use in ancillary studies, if applicable	NA

*It is strongly recommended that this checklist be read in conjunction with the SPIRIT 2013 Explanation & Elaboration for important clarification on the items. Amendments to the protocol should be tracked and dated. The SPIRIT checklist is copyrighted by the SPIRIT Group under the Creative Commons "Attribution-NonCommercial-NoDerivs 3.0 Unported" license.

For peer review only
